# Sensitive dLight3 for imaging broad-spectrum dopamine events across brain regions

**DOI:** 10.21203/rs.3.rs-7313638/v1

**Published:** 2025-08-20

**Authors:** Jacob I. Roshgadol, Julie A. Chouinard, Shouvik Majumder, Erin C. Scott, Katharine Borges, Kenta M. Hagihara, Nino Mancini, Tanner Steveson, Tarun Kamath, Bart Lodder, Bryan J. MacLennan, Rochelin Dalangin, Nikki Tjahjono, Akash Pal, Carina Soares-Cunha, Patrick R. Melugin, Aaron Marley, Karan Mahe, Kiyoto Kurima, Sakiko Takahashi, Dvyne Nosaka, Kazuma Murakami, Lesley A. Colgan, Peter T. Freitas, Rishidev Chaudhuri, Cody A. Siciliano, Ana João Rodrigues, Viviana Gradinaru, Mark Von Zastrow, Kaspar Podgorski, Bernardo L. Sabatini, Salil S. Bidaye, Timothy D. Hanks, Na Ji, Jeffery R. Wickens, Hidehiko K. Inagaki, Lin Tian

**Affiliations:** 1 Max Planck Florida Institute for Neuroscience, Jupiter, FL, USA; 2 Biomedical Engineering Graduate Group, University of California Davis, Davis, CA, USA; 3 Department of Biochemistry and Molecular Medicine, University of California Davis, Davis, CA, USA; 4 Department of Neuroscience, University of California Berkeley, Berkeley, CA; 5 Allen Institute for Neural Dynamics, Seattle, WA, USA; 6 Neuroscience Graduate Group, Center for Neuroscience, University of California Davis, Davis, CA; 7 Department of Neurobiology, Howard Hughes Medical Institute, Harvard Medical School, Boston, MA, USA; 8 Centre de recherche CERVO, Québec, Canada; 9 Life and Health Sciences Research Institute (ICVS), School of Medicine, University of Minho, Braga, Portugal; 10 Department of Pharmacology, Vanderbilt Brain Institute, Vanderbilt Center for Addiction Research, Vanderbilt University, Nashville, TN, USA; 11 Department of Psychiatry, University of California San Francisco, San Francisco, CA, USA; 12 Division of Biology and Biological Engineering, California Institute of Technology, Pasadena, CA, USA; 13 Okinawa Institute of Science and Technology Graduate University, Okinawa, Japan; 14 Center for Neuroscience & Department of Neurology, University of California Davis, Davis, CA; 15 Department of Physics, University of California, Berkeley, CA; 16 Molecular Biophysics and Integrated Bioimaging Division, Lawrence Berkeley National Laboratory, Berkeley, CA, USA

## Abstract

Dopaminergic neurons modulate movement, motivation, and learning by dynamically regulating dopamine release across distributed neural circuits. However, existing genetically encoded dopamine sensors lack the sensitivity and resolution to capture the full amplitude and temporal complexity of *in vivo* dopamine signaling, limiting insight into its functions across behavioral contexts. Here, we present dLight3.8, a fluorescence-intensity and lifetime-based sensor with a substantially expanded dynamic range compared to existing dopamine sensors, enabling transformative detection and differentiation of dopamine release across brain regions and behaviors. Specifically, the enhanced sensitivity of dLight3.8 permits robust, single-trial recording of dopamine release spanning a wide concentration range in response to electrical, optogenetic, and behavioral stimuli, in multiple species and circuits. Using dLight3.8, we uncover a region-specific, gradual shift in dopamine encoding across motor learning, from tracking lick timing to signaling reward prediction. Our findings demonstrate that dLigth3.8 provides quantitatively reliable, highly sensitive measurements of graded dopamine release, which is essential for elucidating diverse roles of dopamine signaling in shaping animal behavior.

## Introduction

Dopamine (DA) is an evolutionarily conserved neuromodulator that plays a central role in shaping behavior across species. Midbrain dopaminergic neurons fire in varying patterns and project broadly to a wide range of brain regions, where they modulate synaptic plasticity, neuronal excitability, and circuit dynamics. Through this widespread influence, DA enables the brain to flexibly adapt to environmental stimuli, supporting behaviors such as movement, foraging, reinforcement learning, and decision-making^1–22^. Altered dopaminergic signaling results in the neurologic dysfunction of diseased states, including Parkinson’s disease, attention deficit hyperactivity disorder (ADHD), and schizophrenia. Many current treatments act by mimicking or globally blocking DA transmission, but they often show limited efficacy and cause undesirable side effects^23–28^. Therefore, developing a comprehensive mechanistic understanding of DA signaling is essential for advancing our ability to design effective treatments.

Technological advancements in large-scale neuronal recording, such as Neuropixels and calcium imaging, have permitted chronic recording of dopaminergic neuronal activity, revealing moment-to-moment changes in regulating action across learning^1,2,7,29,30^. Additionally, genetically encoded DA indicators, such as the dLight and GRAB-DA biosensor families, now enable direct measurement of DA release by averaging trial-aligned signals across behavioral tasks^31–35^. The rapid adoption of these tools has facilitated testing foundational theories, such as reward-prediction error signaling for reinforcement learning, while uncovering novel insights into DA’s role in encoding motivation, reinforcement, and responses to rewarding and aversive stimuli^1,7,12,14,15,17,30^.

However, the fluorescent dynamic range of current DA sensors lags that of modern calcium and glutamate indicators, limiting detection of DA across the broad spectrum of DA release amplitudes^36,37^. For example, DA release measured by voltammetry has shown that first-generation dLight lacked the capacity to accurately detect changes in DA transients elicited by inhibition of DA transporters (DAT)^38,39^. This discrepancy highlighted the sensor’s limitation in dynamic range to reveal the large range of changes in amplitude of DA release under pharmacological modulations. In addition, the low signal-to-noise ratio prevents the precise measurement of small relative changes in DA amplitude, particularly behaviorally modulated changes on a trial-by-trial basis or in brain regions beyond striatum that receive sparse projections^40–43^. As a result, the link between DA neuron activity and downstream release dynamics, that may vary from region-to-region, especially during gradual experience-dependent behavioral adaptations, remains poorly understood. Thus, there is an urgent need for tools that can track moment-to-moment changes of DA release across a wide range of amplitudes to advance mechanistic understanding and therapeutic development.

Here we introduce two dLight3 variants, dLight3.6 and dLight3.8, with distinct but complementary properties, engineered through structure-based mutagenesis and cell-based screening. Compared to the parent sensor dLight1.3b, dLight3.6 displayed 6x tighten apparent affinity and dLight3.8 showed 5x increased dynamic range. We performed comprehensive benchmarking experiments of both variants against dLight1 or GRAB-DA sensors in acute slices and *in vivo* across multiple brain regions and species with various imaging modalities. These include recordings in brain regions that receive sparse DA innervation like the superior colliculus (SC), hippocampus (HPC), and prefrontal cortex (PFC), as well as highly innervated striatal regions. Highlighting the capabilities of the improved sensors, two-photon imaging in SC revealed DA release that was missed by existing DA sensors in response to sensory stimuli.

Among all DA sensors, dLight3.8 with the largest dynamic range enables high-fidelity detection and distinction of DA transients across diverse experimental conditions. In striatal slices, dLight3.8 captured transporter inhibitor–evoked DA signals with amplitudes comparable to fast-scan cyclic voltammetry. Dual-fiber photometry uncovered region-specific and task-specific DA dynamics, with up to a 50-fold difference across regions and a 10-fold difference within the same region. Though the relatively higher apparent affinity of dLight3.6 made it comparable to dLight3.8 in regions with sparse dopaminergic projections, it is less optimal in the striatum, where dLight3.8 performs better. Additionally, dLight3.8 enabled simultaneous fluorescence intensity and lifetime recording *in vivo*, and its sensitivity permitted single-trial resolution of learning-dependent shifts in DA coding, from lick timing to reward anticipation, in a region-specific manner. Thus, the advances introduced with the large dynamic range of dLight3.8 expand the utility of genetically encoded DA sensors, establishing a high-fidelity platform for quantitatively measuring DA release across species and brain regions, and enabling deeper insight into the roles of DA signaling in complex behavior.

## Results

### Engineering dLight3

**Figure 1.**
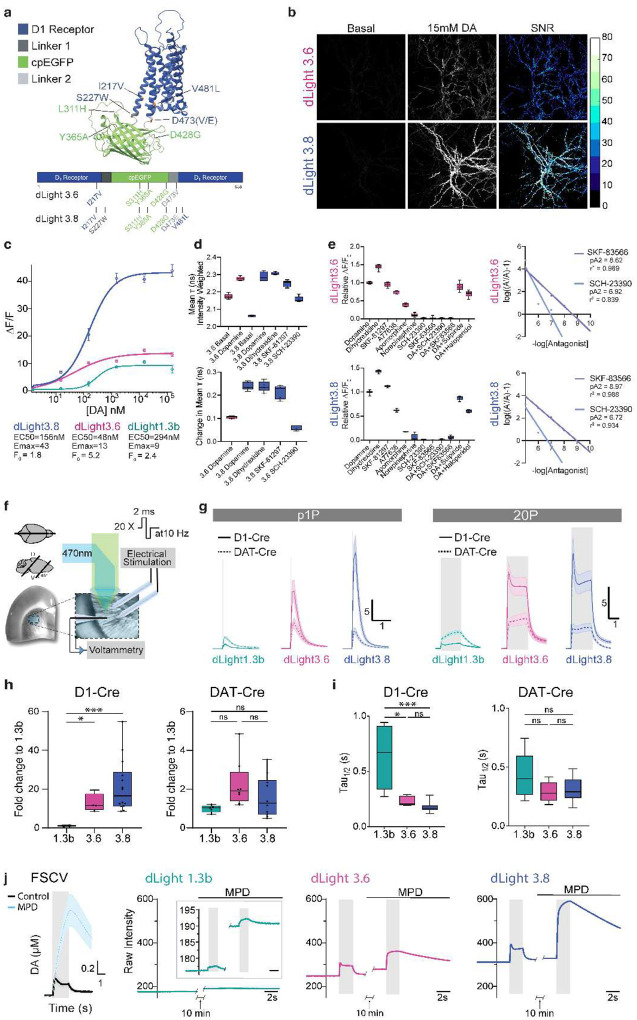
dLight3 Engineering and benchmarking to FSCV in acute brain slice. **a,** Simulated structure using Alphafold of dLight 3.6 consisting of DRD1 (blue), linkers (grey), and cpGFP module (green). Sequence alignment and highlighted amino acid residue changes of dLight 3.6 and 3.8 from parent dLight 1.3b. **b,** Representative images of dLight 3.6 and dLight 3.8 *in vitro* hippocampal neuronal culture. Fluorescence intensity at 15mM DA saturation state and signal-to-noise ratio of apo and saturation state shown. Scale bars = 50 μm. **c,** *in vitro* hippocampal neuron culture dopamine dose-response curves. Data were fitted with Hill Equation, n=4. All data shown as mean ΔF/F_0_ ± SEM. **d,** Mean Tau and Delta Tau lifetime measurements of dLight3.6 and dLight3.8 using HEK T-Rex stable cell line under Dopamine, DRD1 agonists Dyhydrexidine and SKF-81297, and DRD1 antagonist SCH-23390 n=5. **e,** (Left) Pharmacological specificity of dLight 3.6 and dLight 3.8 in HEK T-REx 293 stable cell lines relative to Dopamine response (1.00 ±0.01 ΔF/F_0_, n=8). DRD1 full agonist (Dihydrexidine, 1.43 ± 0.01 ΔF/F_0_, n=8) DRD1 partial agonists (SKF-81297, 1.12 ± 0.01, n=8, A77636, 0.63 ± 0.01, n=8, Apomorphine, 0.17 ± 0.002, n=8), Norepinephrine (0.05 ± 0.02, n=8), DRD1 antagonists (SCH-23390, 0.018 ± 0.001, n=8, SKF-83566, 0.002 ± 0.001, n=8), DA addition following DRD1 antagonists (DA+SCH-23390, 0.020 ± 0.002, n=8, DA+SKF83566, 0.06 ± 0.01, n=8), DRD2 antagonists (Sulpiride, 0.87 ± 0.02, n=8, Haloperidol, 0.60 ± 0.01, n=8). All data shown as mean ΔF/F_0_ ± SEM relative to ΔF/F of dopamine response. All additions at 1μM final concentration. (Right) Combined Schild regression with SKF-83566 and SCH-23390 on dLight 3.6 and dLight 3.8, n=6. **f,** (Top Left) Schematic for DLS slices with most fiber tracks intact. (Right) FSCV setup for electrical stimulation and epifluorescence imaging. **g,** Time-lapse of fluorescent intensity changes (ΔF/F) in response to a pseudo-one-pulse (p1P) or a 20 pulses electrical stimuli in DLS acute slice when expressing dLight at DAT terminals (DAT-Cre expression, dotted line, n = 16, 9, 12 slices from ≥ 3 animals) and postsynaptic cells expressing DRD1 (DRD1-Cre expression, solid line n = 7, 4, 14 slices from ≥ 3 animals). All data shown as mean ΔF/F_0_ ± SEM. **h,** ΔF/F fold change normalized to dLight1.3b. Box plot center line shows the median; box limits show upper and lower quartiles. **i,** Off-rate kinetic Tau_1/2_ at 36.5°C±0.5°C. Epifluorescence imaging at 10 Hz. Violin plot shows the full range of the data, with each line denoting each quartile. **j,** Left: FSCV measurements in DLS acute brain slices without methylphenidate (MPD) (solid black line) and following bath application of MPD (30μM) (dotted blue line). Right: Example traces of time-lapse imaging across the experiment.

To address the limitations of existing dopamine sensors, which consist of a dopamine receptor scaffold coupled to a fluorescent protein that increases in brightness upon ligand-induced conformational change, our engineering efforts focused on dLight1.3b, a human D1 receptor (DRD1)-based sensor with the highest dynamic range of the dLight1 series^31^. To improve dynamic range, we strategically identified specific residues contributing to high basal brightness by comparing the protein structure of two published sensors: dLight1.3b and GRAB_Ach_3.0^31,44^. This revealed six residue positions that are shared between the two sensors, which we hypothesized were critical for high basal brightness: Leu311 and Ser326 in the linker regions, Tyr365 and Asp428 at β-barrel loops, and Lys247 and Ile217 at the cpEGFP-hDRD1 interface (Fig. 1a, **Extended Data Fig. 1a,b,j**). These efforts yielded four mutations: Leu311His, Tyr365Ala, Asp428Gly and Ile217Val (Fig. 1a, **Extended Data Fig. 1a,b,j**). We further optimized the linker residues (Ser227 and Asp473) and key residues on hDRD1 (Tyr218 and Val481) (Fig. 1a, **Extended Data Fig. 1a,b,j**), identified from previous mechanistic studies and GPCR sensor development to have receptor-fluorescent protein interactions, resulting in two final variants, dLight3.6 and dLight3.8 (Fig. 1a, **Extended Data Fig. 1a,b,j**)^44–47^. Specifically, Asp473Val yielded dLight3.6, and Ser227Trp, Val481Leu and Asp473Glu mutations for dLight3.8 (Fig. 1a, **Extended Data Fig. 1b,j)**.

Following genetic engineering, we assessed the performance of these new variants in dissociated neurons (Fig. 1b). Both sensors showed excellent membrane localization and increased DA-bound brightness, with dLight3.6 showing increased basal brightness (Fig. 1b,c, **Extended Data Fig. 2d**). dLight3.6 displayed 1.5x increased dynamic range, 2x increased DA-bound brightness, and 6x tighter apparent affinity (ΔF/F0 _max_ = 13, and EC_50_ = 4 nM), compared to dLight1.3b (Fig. 1c, **Supplementary Table 2**). Additionally, dLight3.8 displayed 5x enhanced dynamic range and ~2x increased apparent affinity (ΔF/F0 max= 43 and EC50 = 156 nM), compared to dLight1.3b (Fig. 1c, **Supplementary Table 2**). The sensitivity represented by s-slope, the maximum slope of the sensor’s dose-response curve, for both dLight3.6 and dLight3.8 is increased by 10-fold compared to dLight1.3b, (S-slope(uM^−1^) dLight3.6 = 27, dLight3.8 = 29, and dLight1.3b = 2.7; **Supplementary Table 2**)^48^.

Next, we engineered cell lines stably expressing dLight3 variants to ensure long-term and uniform protein expression during characterization of sensor activity in response to a panel of DA receptor-targeting drugs (Fig. 1e)^49^. We determined the excitation (Ex max=490–492nm) and emission (Em max=500–510nm) peaks and isosbestic point (Iso dLight3.6=406nm and Iso dLight3.8=419nm) of dLight3.6 and dLight3.8 to guide optimal imaging parameters (**Extended Data Fig. 1h,i**). Both dLight3 variants showed similar responses to the drugs relative to the sensors’ response to DA, with the largest response to a full D_1_ agonist, dihydrexidine, and reduced responses to SKF-81297, A77636 and apomorphine, which are partial D_1_ agonists (Fig. 1e). We also examined fluorescence lifetime changes of dLight3.6 and dLight3.8 in response to various agonists^50,51^. The lifetime change of dLight3.8 to DA (ΔT=240ps) is similar to dihydrexidine (ΔT=238ps), and dLight3.8 shows higher lifetime change to DA does dLight3.6 (ΔT=105ps; Fig. 1d). The presence of D_1_ antagonists SKF-83566 and SCH-23390 did not evoke a response on their own and abolished responses elicited by DA, whereas D_2_ antagonists only slightly impaired the DA-evoked response. D_1_ antagonists SKF-83566 showed stronger inhibition on sensors than SCH-23390 (Fig. 1e, **Extended Data Fig. 2b, Supplementary Table 3**). In addition, dLight3.6 and dLight3.8 do not affect endogenous D1 receptor signaling, as evidenced by their failure to induce cAMP or recruit β-arrestin in response to SKF-81297 (**Extended Data Fig. 1e-g**).

Given the structural similarity between different neuromodulators and their tendency to bind to each other’s receptors, we examined the selectivity of both sensors to other neuromodulators such as serotonin (SE) and norepinephrine (NE) (**Extended Data Fig. 2c,e-g, Supplementary Table 4,5**)^52,53^. Only negligible responses were observed to SE in stable HEK cells expressing dLight3 (**Extended Data Fig. 2f, Supplementary Table 4)**. In neurons, dLight3.6 and dLight3.8 showed ~60 fold less sensitivity to NE than DA respectively (**Extended Data Fig. 2c,e, Supplementary Table 2**). In HEK cells stably expressing the sensor, increasing the concentration of NE shifted the apparent affinity of dLight3.6 for DA, whereas dLight3.8 binding to DA remained largely unaffected until very high NE concentrations (100 μM), suggesting that dLight3.8 exhibits greater selectivity for DA compared to dLight3.6 (**Extended Data Fig. 2g, Supplementary Table 4,5)**. These *in vitro* characterizations indicate that dLight3.6 and dLight3.8 successfully address key limitations of earlier dLight1 sensors, exhibiting markedly improved dynamic range and enhanced affinity for DA.

### Benchmarking dLight3.6 and dLight3.8 in acute striatal slice with cell-type specificity

To assess whether dLight3’s enhanced *in vitro* performance extends to more physiological conditions, we tested its ability to detect electrically evoked DA release in acute striatal slices, comparing pre- and postsynaptic expression in the dorsolateral striatum (DLS) (Fig. 1f–j). We used a Cre-dependent dual promoter system (hSyn-tTA/TRE-DIO) to drive dLight expression, as this promoter improved the brightness of sensor expression compared to the CAG promoter in DLS (**Extended Data Fig. 3a**). AAV viruses (AAV.Syn-tTA/TRE-DIO-dLight3.6, 3.8 or 1.3b) were injected either in the DLS of DRD1-Cre mice or in the substantia nigra pars compacta (SNc) of DAT-Cre mice, followed by wide-field imaging in DLS with electrical stimuli. In DLS acute slice of DRD1-Cre animals, dLight3.6 and dLight3.8 showed ~14 and ~20 fold increased dynamic range, respectively, to pseudo-one pulse (p1P) electrical stimuli compared to dLight1.3b (dLight1.3b: ΔF/F =1.20±0.2%,; dLight3.6: ΔF/F =16.8±3.4%, dLight3.8: ΔF/F=23.6±4.0%; Fig. 1g,h, **Supplementary Table 6**). Increased electrical stimuli (20P) led to sustained release but did not increase the peak amplitude of any of the sensor variants. (Fig. 1g,h, **Supplementary Table 6**). The decay time from peak to baseline in response to p1P is ~3x and ~4x faster for dLight3.6 and dLight3.8, respectively, compared to dLight1.3b (Fig. 1i, **Supplementary Table 6**). In DLS slice of DAT-Cre animals, however, the responses of dLight3.6 and dLight3.8 were significantly reduced, and are only slightly higher compared to dLight1.3b (dLight1.3b : ΔF/F=2.8±0.2%; dLight3.6: ΔF/F=5.7±1.2%,; dLight3.8: ΔF/F=4.3±0.8%; Fig. 1g,h, **Supplementary Table 6**). The decay time from peak to the baseline in response to p1P for all three sensors is similar in DLS slice of DAT-Cre animals (Fig. 1i, **Supplementary Table 6**).

### Benchmarking dLight3.6 and dLight3.8 with Fast-Scanning Cyclic Voltammetry

Given the significantly larger response of dLight3.8 in DRD1-expressing cells, we next benchmarked the response of dLight3.8 to Fast Scanning Cyclic Voltammetry (FSCV) using DLS acute slice from DRD1-Cre animals. Previous research has shown distinct waveforms between dLight1.1 and FSCV measurements of electrically evoked DA responses^39^. As the linear range of fluorescence changes of single-FP based sensor depends on basal ligand concentration and EC50, we wanted to ask if the improved dynamic range offered by dLight3.8 would enable detection of comparable waveforms of DA release as measured by FSCV during electrically triggered DA release in the presence of a DAT blocker (Fig. 1j, **Extended Data Fig. 3b-e**)^54^. In acute DLS slice of DRD1-Cre animals, simultaneous time-lapse wide-field imaging and FSCV revealed that is similar to dLight1.1, dLight1.3b and dLight3.6 fail to replicate the waveform of electrically evoked DA release measured with FSCV in the presence of DAT blocker methylphenidate (MPD) (Fig. 1j, **Extended Data Fig. 3d,e, Supplementary Table 6**). In contrast, dLight3.8 showed increased baseline fluorescence, significantly increased peak amplitude and duration of transients comparable to the voltammetry waveform (Fig. 1j, **Extended Data Fig. 3e, Supplementary Table 6**). This *ex vivo* results demonstrate that the superior dynamic range of dLight3.8 is critical for resolving stimulus-evoked changes in DA release under pharmacological modulations, including both baseline shifts and amplified release dynamics in the presence of elevated extracellular DA caused by a DAT blocker.

### Two-photon imaging of DA release using engineered AAVs across brain regions in acute brain slice

Recently engineered AAVs that can cross the blood-brain barrier have been broadly used to target genetically encoded markers throughout the brain with a single, minimally invasive intravenous injection^55–58^. However, such noninvasive, systemic delivery has been challenging when combined with membrane- targeted sensors and effectors. To date, only highly optimized calcium indicators such as GCaMPs have been delivered and characterized using these novel engineered AAVs^55,56^. We thus sought to characterize the expression and responses of dLight3 across brain regions via systemic injection of newly developed engineered AAVs.

**Figure 2.**
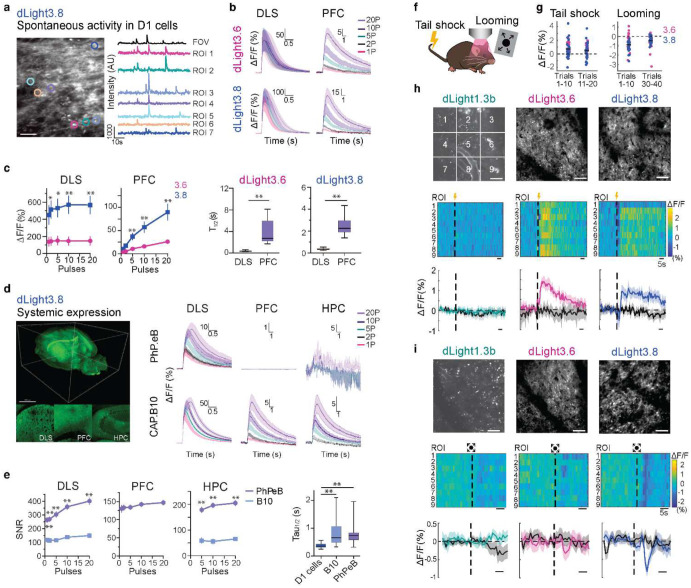
Two-Photon imaging of dLight3 in acute brain slices and *in vivo* in superior colliculus. **a-f,**Two-photon imaging of dLight3 in acute slice**. a,** Spontaneous dLight3.8 activity revealed by two-photon imaging displayed spatial heterogeneity, timelapse of fluorescence intensity from the identified ROIs in DLS acute slice. Scale bars: 10μm. **b,** Time-lapse of fluorescent intensity changes (ΔF/F) in response to electrical stimuli (1, 2, 5, 10 or 20 pulses, 40Hz) in DLS (n=13 for 3.6, n=18 for 3.8) and PFC (n=12 for 3.6, n=6 for 3.8), following stereotaxic injection. Mean ΔF/F_0_ ± SEM. **c,** (Top) Maximum peak amplitude in response to 1, 2, 5, 10 or 20 pulses for each sensor. (Bottom) Off-rate kinetic Tau_1/2_ at 29°C. Box plot center line shows the median; box limits show upper and lower quartile. **d,** (Top Left) Cleared brain showing dLight3.8 expression across brain regions following systemic injection of CAP-B10 viral capsid. Scale bar: 3000μm. (Bottom Left) Representative images of DLS, PFC, and HPC of dLight3.8 expression. Scale bar: 100μm. (Right) Time-lapse of fluorescent intensity changes (ΔF/F) in response to electrical stimuli in acute slices following 6 weeks expression of systemic injection using either PhP.eB or CAP-B10 viral capsid. DLS (PhP.eB n=12, B10 n=31), PFC (PhP.eB n=3, B10 n=14) and HPC (PhP.eB n=3, B10 n=18). Mean ΔF/F_0_ ± SEM. **e,** (Top) SNR of peak amplitude increased with the number of stimuli, comparing CAB-B10 and PhP.eB. (Bottom) Peak ΔF/F and Tau_1/2_ at 20Hz comparing systemic delivery and stereotaxic injection. Box plot center line shows the median; box limits show upper and lower quartile. **f-i,** *In vivo* two-photon imaging of dLight3 benchmarked to dLight1.3b. **f,** Schematic of tail shock and looming behavior paradigm **g,** (Left) Trial-averaged peak responses for the first 10 shock trials versus the last 10 shock trials. Response amplitude is defined as the average ΔF/F between 3.2–5.5 s after shock onset across trials. Each symbol represents one ROI within a field of view. Black bars: mean. ***P<0.001 (paired t test). (Right) Trial-averaged peak responses for the first 10 looming trials versus the last 10 looming trials. Each symbol represents one ROI within a field of view. Black bars: mean. ***P<0.001 (paired t test). **h,** (Top) averaged two-photon images of dLight3.6, dLight3.8, and dLight1.3b expressed in SC. Grid lines superimposed on the dLight1.3b image divide the field of view into 9 ROIs. Scale bar: 50μm. (Middle) Heatmap of trials averaged ΔF/F_0_ (20 trials) to tail shock for dLight3.6, dLight3.8, and dLight1.3b for each ROI. Dashed lines: onset of 0.5-s-long shock. Scale bar: 5sec. (Bottom) Trial-averaged ΔF/F_0_ (mean ± SEM) for the entire field of view. Black lines with gray shading: trial-average without shock. Scale bar: 5sec. **i,** (Top) averaged two-photon images of dLight1.3b, dLight3.6, and dLight3.8 expressed in SC. Scale bar: 50μm. (Middle) Heatmap of trials averaged ΔF/F_0_ (40 trials) to looming stimuli of dLight1.3b, dLight3.6, and dLight3.8 for ROIs. Dashed lines: onset of 1-s-long looming. Scale bar: 5sec. (Bottom) Trial-averaged ΔF/F0 (mean ± SEM) for the entire field of view. Black lines with gray shading: Trial-average without looming. Scale bar: 5sec.

To benchmark systemic delivery to direct stereotaxic delivery, we first injected AAV viruses (hSyn-tTA/TRE-DIO-dLight3.6 or 3.8) in DLS and PFC of DRD1-Cre mice followed by two-photon imaging in acute brain slice. In DLS, we observed spontaneous DA release with dLight3.8 (Fig. 2a). At one field stimulus, dLight3.8 displayed ~3x increased peak amplitude compared to dLight3.6 in DLS (ΔF/F=49 ± 111%, ΔF/F=138 ± 53%); the peak amplitude for both sensors only slightly increased with the increased number of stimuli (Fig. 2b,c, **Supplementary Table 6**). In PFC, we observed increased peak amplitude with the increased number of stimuli for both sensors (Fig. 2b,c, **Supplementary Table 6**); dLight3.8 displayed ~3.5 fold increased amplitude compared to dLight3.6 in response to 20 field stimuli (ΔF/F=89.5 ± 17.5%, dLight3.8; ΔF/F=26.2 ± 3.5%, dLight3.6, **Supplementary Table 6**). Both sensors displayed significantly increased peak amplitude and faster off kinetics (~10x) when expressed in DLS compared to PFC (Fig. 2c, **Supplementary Table 6**). This slower off rate in PFC suggests that DA clearance in the PFC may be slower due to the low expression of DA transporters^42,59–61^.

As dLight 3.8 outperformed dLight3.6 in both DLS and PFC, we next compared the utility of two novel blood-brain barrier crossing engineered AAVs, AAV-PHP.eB and AAV.CAP-B10 in expressing dLight3.8. Six weeks after tail vein injection, while the effective dynamics of dLight3.8 was limited to DLS and partially in HPC when AAV-PHP.eB was used, widespread effective dynamics were observed across brain regions using AAV.CAP-B10 (Fig. 2d,e, **Extended Data Fig. 4, Supplementary Table 6**)^55,58^. We next performed two-photon imaging in brain slice to examine the responses of dLight3.8 to electrical stimuli, selectively in DLS, PFC and HPC. dLight3.8 showed robust responses to increased number of electrical stimulations in all three regions (Fig. 2d,e, **Extended Data Fig. 4b, Supplementary Table 6**) with CAP-B10. In DLS, the peak amplitude of dLight3.8 expressed via CAP-B10 is significantly higher (~ up to 5x) than that via PHP.eB (Fig. 2d,e, **Extended Data Fig. 4b, Supplementary Table 6**). The SNR of dLight3.8 responses via PhP.eB were significantly reduced in HPC and remain undetectable in PFC (Fig. 2d,e, **Extended Data Fig. 4b, Supplementary Table 6**). Taken together, the *ex vivo* results suggest dLight3.8 permits robust two-photon imaging of DA release across brain regions triggered by electrical stimuli via systemic delivery.

### Two-photon imaging of fast and sustained DA release in SC of awake mice

Given high *ex vivo* performance of both dLight3.6 and dLight3.8, we next aimed to benchmark dLight3.6 and dLight3.8 *in vivo* using two-photon imaging in the SC, where DA detection had previously failed with dLight1.3b. The SC, a midbrain structure involved in detecting and responding to salient stimuli, receives sparse but significant DA input from regions like the zona incerta and locus coeruleus^62^. While dLight1.3b (AAV9-CAG-dLight1.3b) failed to detect DA release to either aversive tail shocks (Fig. 2f,h) or naturalistic looming stimuli (Fig. 2f,i), both dLight3.6 and dLight3.8 reliably reported robust DA signals in SC in response to both stimuli^63–66^.

Shocks elicited a sustained fluorescence increase lasting tens of seconds in both dLight3.6 and dLight3.8 variants (Fig. 2h). To investigate spatial heterogeneity in DA release, we divided each field of view (FOV) into a 3 × 3 grid, yielding nine regions of interest (ROIs; Fig. 2h). Trial-averaged ΔF/F traces revealed considerable variability across ROIs (Fig. 2h): some regions exhibited brief transients (e.g., Fig. 2h, ROI 9), others showed sustained responses (e.g., Fig. 2h, ROI 2), while several regions displayed minimal or no detectable signal (e.g., Fig. 2h, ROIs 5 and 7). Next, we asked whether the amplitude of fluorescence transients changed over the course of repeated shock trials (Fig. 2g **left**). Late-trial responses were modestly but significantly reduced relative to early trials, suggesting a decline in DA release with repeated stimulation (Fig. 2g **left**, mean ΔF/F₀: 0.712% early vs. 0.493% late; P = 0.0129, one-sided paired *t*-test).

In contrast to the shock stimulus, the looming stimulus led to a decrease in fluorescence, indicating a suppression of DA release lasting approximately 1 – 2 s (Fig. 2i). dLight3.8 consistently reported decreases in fluorescence across all ROIs, whereas dLight3.6 detected decreases in only a subset of ROIs (Fig. 2i). In addition, late-trial responses were significantly attenuated (−0.891% early vs.−0.4621% late; P = 1.06e-4, one-sided paired t-test), indicating suppressed DA release with repeated exposure (Fig. 2g **right**).

These results suggest that the improved sensitivity of dLight3 variants, especially dLight3.8, not only permits robust detection of DA in regions receiving moderate input like the SC but also provides crucial insights into the potential mechanisms by which DA modulates saliency detection.

### Benchmarking dLight3 responses to optogenetic stimuli in mice and flies

**Figure 3.**
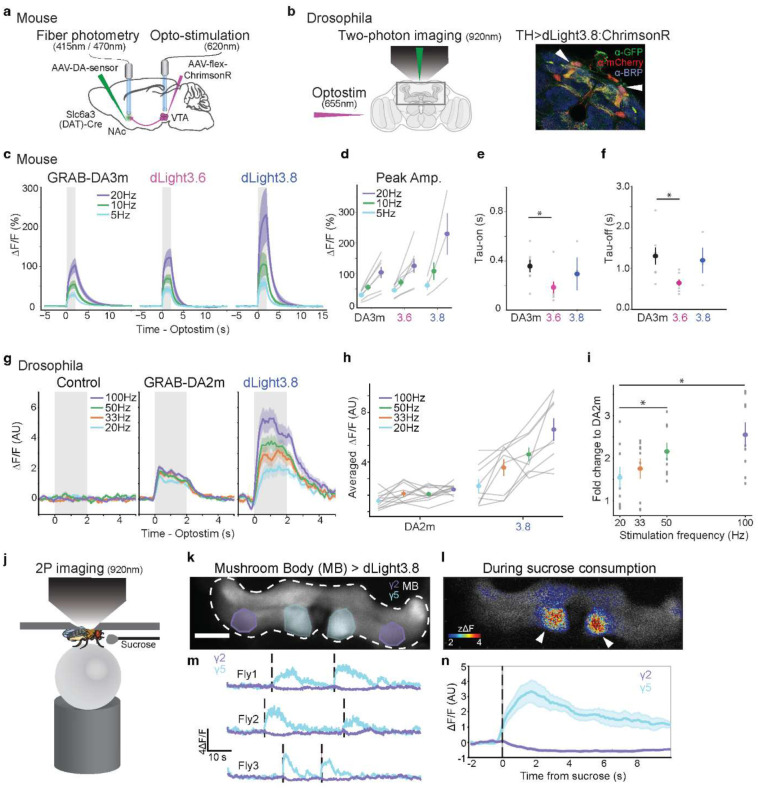
Benchmarking dLight3.0 to GRAB-DA using optogenetic stimuli in mice and flies. **a,** Schematic showing dLight expression in NAc and ChrimsonR expression in VTA of DAT-Cre mice as well as fiber implantation for simultaneous optogenetic stimulation and recording. (GRAB-DA3m, n=7; dLight 3.6, n=7; dLight3.8, n=5). **b,** Schematic of the experimental design consisting of simultaneous two-photon imaging and widefield optogenetic stimulation of TH-Gal4 neurons co-expressing a dopamine sensor and ChrimsonR::mCherry in explant fly brains. Yellow square shows mushroom body neuropil. Immunochemistry images show co-staining of dLight3.8 (green), mCherry (red) and neuropils (blue). White arrowheads show TH-labeled neurons innervating the mushroom body vertical lobes. Scale bar: 50 μm. (GRAB-DA2m, n=12; dLight3.8, n=9). **c,** ΔF/F_0_ mean ± SEM traces time-series of GRAB-DA3m, dLight3.6, and dLight3.8 to 40 trials of repeated optogenetic stimulation of ChrimsonR at 5, 10, and 20Hz stimulation frequencies. **d,** Average peak amplitude of the dopamine sensors to the three different optogenetic stimulation frequencies. **e,f,** Tau on and off of the dopamine sensors to optogenetic stimulation. Tau-on (s) DA3m vs 3.6: p = 0.037, Tau-off (s) DA3m vs 3.6: p = 0.018 determined by t-test. **g,** Dopamine signal from TH-labelled neurons innervating the mushroom body vertical lobe expressing either dLight3.8 only (left), GRAB-DA2m with ChrimsonR::mCherry (middle) or dLight3.8 with ChrimsonR::mCherry (right). Grey bars show 2s optogenetic stimulation delivered at different frequencies. Traces show ΔF/F averaged across brains, mean ± SEM. **h,** Peak ΔF/F at different stimulation frequencies, calculated from g. Grey lines show individual brains; colored dots show mean ± SEM n=9–12 brains per genotypes. **i,** Fold change to GRAB-DA2m at different stimulation frequencies, calculated from h. Asterisks indicate significant differences between groups based on paired t-tests with Bonferroni correction (p < 0.0083). **j-n,** Two-photon imaging of dopamine in behaving flies **j,** Schematic of a head-fixed fly walking on an air-suspended ball while receiving 1 M sucrose solution, with dLight3.8 signal imaged in the mushroom bodies using two-photon microscopy. **k,** Two-photon maximum intensity projection of dLight3.8 fluorescence in the mushroom bodies (white dashed outlines); γ2 and γ5 compartments are shaded in blue and magenta, respectively. Scale bar: 10 μm. **l,** dLight3.8 signal (zΔF) during sucrose consumption, averaged across sucrose presentations in an individual fly; white arrowheads indicate γ5 compartments. **m,** Example traces of dLight3.8 signal from γ2 and γ5 compartments during sucrose consumption in three individual flies; dashed lines indicate feeding onset. **n,** dLight3.8 signal (ΔF/F: mean ± SEM) in γ2 and γ5 compartments during sucrose consumption, averaged across sucrose presentation and flies (n = 6); red bar indicates feeding onset.

After *ex vivo* and *in vivo* characterization demonstrated a reliable increase in dLight3 performance compared to the parent sensor, dLight1.3b, we next sought to benchmark dLight3 sensors to other genetically encoded DA indicators across species. Optogenetics triggers controlled and reproducible DA release, making it a robust and valid approach for cross-sensor comparisons. We thus characterized the responses of dLight3 variants and GRAB-DA sensors in mice via fiber photometry (Fig. 3a) and in *Drosophila melanogaster* via two-photon imaging (Fig. 3b).

In DAT-Cre mice, DA indicators (AAV-CAG vectors carrying GRAB-DA3m, dLight3.6 or dLight3.8) were virally transduced to the nucleus accumbens (NAc), and ventral tegmental area (VTA) DA neurons were virally labeled with a red-shifted opsin, ChrimsonR (AAV-CAG-DIO-ChrimsonR) (Fig. 3a). Four weeks later, we used fiber photometry to record DA transients in NAc in response to repeated light stimulations (620nm) in VTA (Fig. 3a). We observed reliable responses over the course of 40 trials of photostimulation with various pulse frequencies (**Extended Data Fig. 5**). The averaged peak amplitude was increased with various pulse train frequencies for all three sensors (Fig. 3c,d), at 5, 10, and 20 Hz stimulation, ΔF/F responses were: GRAB-DA3m: 30.6 ± 5.6%, 56.0 ± 9.6%, 104.4 ± 17.7%; dLight3.6: 46.2 ± 6.9%, 71.9 ± 11.2%, 126.0 ± 22.5%; dLight3.8: 62.2 ± 14.8%, 108.7 ± 28.8%, 230.4 ± 67.5%). Overall, dLight3.8 showed more than 2-fold larger peak amplitude (ΔF/F) compared to GRAB3m (Fig. 3c,d). Though dLight3.6 displayed a slightly larger peak (~1.2 fold) compared to GRAB3m, dLight3.6 exhibited faster on time and decay time (0.64 ± 0.08 s) than GRABDA3m (1.30 ± 0.21 s) and dLight3.8 (1.20 ± 0.31 s) (Fig. 3e,f).

In *Drosophila melanogaster*, dLight3.8 or GRAB-DA2m were co-expressed with the red-shifted opsin ChrimsonR in tyrosine hydroxylase (TH)-positive dopaminergic neurons (Fig. 3b). Two-photon imaging of sensor fluorescence was used to monitor responses to optogenetic stimulation at varying frequencies (20, 33, 50, and 100 Hz, Fig. 3g,h). dLight3.8 exhibited frequency-dependent increases in peak fluorescence amplitude (Fig. 3g,h). Across all tested frequencies, dLight3.8 consistently showed ~1.5–3-fold higher peak amplitude compared to GRAB-DA2m (Fig. 3i).

Beyond evoked response, the high sensitivity of dLight3.8 also enabled two-photon imaging of region-specific detection of DA release in head-fixed walking flies while consuming sucrose (Fig. 3j). Following sucrose delivery, we observed large and sustained increases at single-trial level in the mushroom body Gamma 5 compartment, while the Gamma 2 compartment displayed a slight decrease (Fig. 3k–n). These results are consistent with previous findings of compartment-specific modulation of dopamine during reward-related behavior^67–71^.

### Simultaneous monitoring of DA release dynamics in mouse PFC and NAc during reward seeking and fear learning behavior using dual-fiber photometry

**Figure 4.**
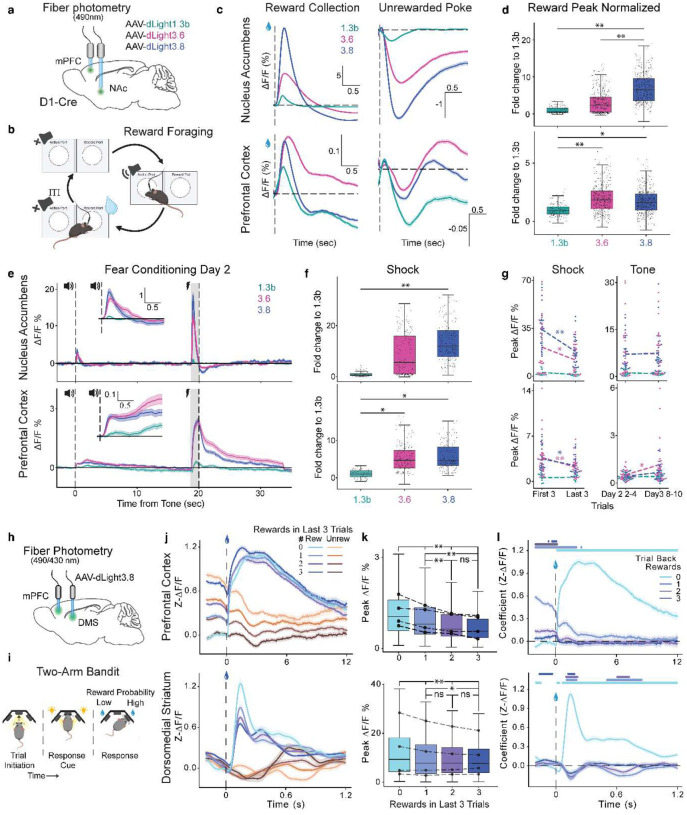
In vivo dual-fiber photometry recordings across brain regions in mice and rats. **a,** Schematic of viral injection of a single dLight variant and dual-fiber-optic implant in NAc and PFC of a mouse brain for sensor benchmarking (dLight3.6, n=9; dLight3.8, n=9; dLight1.3b, n=3). **b-d,** dLight benchmarking during operant reward foraging in mice. **b,** Schematic of the operant reward foraging protocol. **c,** Average dLight response to pokes at the reward port when rewards are collected (left) and when no reward is delivered (right) in NAc (top) and mPFC (bottom). Signals are shifted so the baseline is equal for every trial at the time of the reward delivery. Signals are averaged over all trials and subjects. Shaded areas indicate SEM. **d,** Peak of dLight transient in response to reward collection for all three dLight variants normalized to the average peak reward response of dLight1.3b. Differences between variants were characterized using linear mixed-effects models with a fixed effect for each variant group and a random effect for each subject (NAc, 3.8 vs 1.3b ***: p = 1.3e-5, 3.8 vs 3.6 ***: p = 2.2e-4. PFC, 3.8 vs 1.3b *: p = 0.03, 3.6 vs 1.3b **: p = 5e-3). Box plot center line shows the median; box limits show upper and lower quartiles. Dots show individual trial responses. **e-g,** dLight benchmarking during classical fear conditioning in mice. **e,** Average dLight response on day 2 of fear conditioning in NAc (top) and mPFC (bottom). Inset: response to tone initiation. Signals are shifted so the baseline is equal for every trial at the time of the tone initiation. Signals are averaged over all trials and subjects. Shaded areas indicate SEM. **f,** Peak of dLight transient in response to the shock for all three dLight variants normalized to the average peak shock response of dLight1.3b. In NAc (top) dLight3.8 has 14.3x and dLight3.6 has 9.2x higher response than 1.3b. In PFC (bottom) dLight3.8 and dLight3.6 both have 5.6x higher response than 1.3b. Differences between variants were characterized using linear mixed-effects models with a fixed effect for each variant group and a random effect for each subject (NAc, 3.8 vs 1.3b **: p = 8e-3. PFC, 3.8 vs 1.3b *: p = 0.037, 3.6 vs 1.3b *: p = 0.037). Box plot center line shows the mean; box limits show upper and lower quartiles. Dots show individual trial responses. **g,** dLights 3.8 and 3.6 measure a decreased shock peak transient between trials 1–3 and 18–20 of conditioning (left). dLight3.6 measures an increase in tone response in PFC between the first three trials after the animals experience shock during conditioning and the last three trials of extinction (right). Differences between shock and tone responses were characterized using a linear mixed-effects model with a fixed interaction effect for each variant and trial group and a random effect for each subject. (Shock - NAc, 3.8 ***: p = 3.8e-4, 3.6 *: p = 0.01. PFC, 3.8 *: p = 0.015, 3.6 **: p = 4e-3. Tone - PFC, 3.6 *: p = 0.039). **h-l,** dual-site dLight3.8 recordings in rats during a probabilistic two-armed bandit reward choice task (n=4). **h,** Schematic showing expression of dLight3.8 and dualfiber-optic implant in PFC and DMS of rat brain. **i,** Schematic of the probabilistic two-armed bandit reward choice task. **j,** Average z-scored ΔF/F dLight traces at the time of reward delivery grouped by trial outcome and number of rewards received over the last 3 trials. Signal baselines are shifted for each session based on the average of the signal from the lowest reward condition in the 100ms before reward delivery. Signals are averaged over all sessions and subjects. Shaded areas indicate SEM. **k,** Distributions of reward peak amplitudes of dLight transients on individual trials for each reward history condition. Peak amplitudes were calculated as the difference between peak values within a window of time after reward and the deepest trough on either side of the peak. In both regions, the peak amplitudes decrease as the number of prior rewards increases. Differences between reward history conditions were characterized using linear mixed-effects models with a fixed effect for each reward history group and a random effect for each subject (*: p < 8e-3, **: p < 1e-4, Bonferroni correction at p < 0.05). Box plot center line shows the median, and the box limits are the 1st and 3rd quartile. Connected dots show averages for each subject. **l,** Independent effect of rewards received at each trial-back latency on the reward-related transient of the current trial. Each trace indicates the linear regression coefficient for a reward received at each trial-back latency predicting the z-scored ΔF/F values at each aligned timepoint across all sessions and subjects. The ‘0’ trial-back trace is the regression intercept and captures the average reward transient when there were no rewards in the last 3 trials. Dots above the traces indicate timepoints where the associated coefficient was significantly different than 0 (two-tailed t-test, Bonferroni correction at p < 0.05). Shaded areas indicate SEM.

Building on findings of spatially resolved DA release in head-fixed mice and flies with two-photon imaging, we next sought to investigate how DA is coordinated across multiple brain regions during complex behaviors in freely moving rodents. To do so, we performed dual-fiber recording in the PFC and NAc simultaneously, regions critically involved in reward processing and fear learning^23,38,72,73^. We injected a single variant, dLight1.3b, dLight3.6, or dLight3.8, into both brain regions in opposite hemispheres of DRD1-Cre mice, followed by implantation of a dual fiber-optic cannula (Fig. 4a, **Supplementary Fig. 2**).

Four weeks later, we trained the mice on a naturalistic operant foraging task, in which they performed a series of nose-pokes to earn rewards (Fig. 4b). When animals became experts and completed at least one trial per minute on average, we collected dLight signals from 3–7 sessions with ~300 trials per animal. We observed large transients in both the PFC and NAc when animals collected rewards at the reward port, and smaller decreases in signal when they nose-poked without receiving a reward (Fig. 4c). In the NAc, reward collection triggered robust DA release, with dLight3.6 and dLight3.8 showing 3-fold and 7-fold higher peak responses than dLight1.3b, peak average ΔF/F = 9.7±.2%, 22.1±.2%, and 3.2±0.1% for 3.6, 3.8 and 1.3b respectively (Fig. 4d). In contrast, reward-evoked DA signals in the PFC were substantially smaller, with dLight3.6 and dLight3.8 reaching only 2.3-fold and 1.7-fold higher than dLight1.3b, peak average ΔF/F = 0.37±0.004%, 0.32±0.003%, and 0.19±0.003% for 3.6, 3.8 and 1.3b respectively (Fig. 4d). Despite the reduced amplitude, dLight3 variants reliably detected reward-related DA release even in the low-DA environment of the PFC (Fig. 4c,d).

After a one-week break in their home cages, we measured DA transients during a 3-day classical auditory fear conditioning experiment (Fig. 4e–g). In both NAc and PFC, no tone-evoked response was detected on day 1 (**Extended Data Fig. 6a,c**). During conditioning (day 2), all three sensors showed a small transient at tone onset and a large transient at shock onset (Fig. 4e). Tone-triggered transients remained during extinction (day 3) (Fig. 4e,g, **Extended Data Fig. 6a,c**). The post-shock responses of dLight3.6 and dLight3.8 were significantly higher than dLight1.3b during shock trials: 8.9-fold and 13.9-fold in Nac, peak average ΔF/F = 14.3±1%, 22.2±1%, and 1.6±0.2% for 3.6, 3.8 and 1.3b respectively, and 5.6-fold and 5.6-fold in the PFC, respectively, peak average ΔF/F = 2.7±0.1%, 2.7±0.1%, and 0.5±0.06% for 3.6, 3.8 and 1.3b respectively (Fig. 4f).

Shock-triggered transients also lasted significantly longer in the PFC than in NAc, consistent with *ex vivo* results suggesting slower DA clearance due to lower DAT expression (Fig. 2c, **Extended Data Fig. 6b**)^59–61^. Across conditioning trials, both regions showed a decline in peak amplitude of shock-evoked responses, which can only be detected by both dLight3.6 and dLight3.8 but not dLight1.3b, suggesting improved sensitivity of dLight3 variants and reduced shock novelty (Fig. 4g)^12,41^. In PFC, tone-evoked responses were enhanced as evidenced by dLight3.6 only during extinction, a trend absent in the NAc (Fig. 4g).

Together, these results suggest dLight3 variants enable detection of a broad spectrum of task-specific DA release amplitudes, which can differ drastically across brain regions. In the NAc, dLight3.8 outperformed dLight3.6 in detecting DA transients during both reward and shock. However, the higher affinity of dLight3.6 allowed it to perform comparably to dLight3.8 in the PFC, where dopaminergic projections are sparse and release concentration may be lower^42,43,59–61^.

As behaviorally relevant DA signals have been controversial in PFC, we sought to confirm the results of dual-fiber recording using single-fiber photometry recording^72,74^. Consistent with dual-fiber results, both variants reliably detected DA release in response to shock cues, shock onset, neutral tones, reward collection in the PFC, with dLight3.6 showing the highest amplitude among all variants (**Extended Data Fig. 7**)^42^.

### Simultaneous monitoring of DA release dynamics in rat dorsomedial striatum and PFC during reward seeking using dual-fiber photometry

Based on the region-specific sensitivity of dLight3.8 in mice, we next asked whether dLight3.8 could reliably resolve DA dynamics across circuits in rats engaged in a cognitively demanding task. To do so, we performed dual-fiber recordings in the dorsomedial striatum (DMS) and PFC during a probabilistic reward-choice task (Fig. 4h)^75,76^. In this task, rats initiate trials by nose-poking and holding in the central port. After a variable delay (0.5–1 s), the side ports illuminate to cue a choice. Nose poke in each side port was tied to a reward probability (20–80%), which switched every 25–40 trials (Fig. 4i). Rats consistently favored the higher-probability port and adapted their choices following probability shifts (**Extended Data Fig. 8c**). In well-trained animals, dLight3.8 was expressed in the DMS and PFC in opposite hemispheres (**Extended Data Fig. 8b**). The fluorescence intensity of dLight3.8 signals were recorded, on average, across 6 sessions with ~1800 trials per animal.

In both regions, we detected DA transients after the response cue and reward delivery (Fig. 4j–l). The peak amplitude in DMS were an order of magnitude larger than those in the PFC after both the response cue (9.25 ± 0.1 vs 0.30 ± 0.01 %ΔF/F) and reward delivery (10.6 ± 0.1 vs 0.881 ± 0.01 %ΔF/F) (**Extended Data Fig. 8d-f, top panel**), which is consistent with previous literature^23,30,77^. Additionally, PFC transients were an order of magnitude longer than those in DMS after the response cue (full-width half maximum: 1.12 ± 0.01 vs 0.230 ± 0.001 s) and reward delivery (3.59 ± 0.04 vs 0.225 ± 0.002 s) (**Extended Data Fig. 8d-f, bottom panel**), possibly due to the lack of DAT in PFC^59–61^.

Next, we asked whether dLight3.8 could support trial-by-trial analysis of how reward transients are modulated by prior reward history (Fig. 4j). We found that peak amplitudes decreased as the number of prior rewards increased in both regions, with a stronger effect in the PFC (Fig. 4k). To further quantify the effects of reward history on DA transients, we applied linear regression across multiple trial latencies (Fig. 4l)^78^. In both regions, rewards up to three trials back significantly influenced DA transients, but with distinct patterns: in the DMS, prior rewards directly suppressed the reward-evoked peak (negative regression coefficients 1–3 trials back), whereas in the PFC, prior rewards elevated baseline fluorescence without altering the peak itself, reducing the pre- to post-reward amplitude shift (Fig. 4l). These data show dLight3.8 permits high-fidelity detection of striking, reward-history–dependent changes driven by subtle shifts in DA release. These findings, consistent with dual-fiber recordings in mice, combined with *Drosophila* two-photon recordings suggest the robust cross-species utility of dLight3.8 for monitoring DA release.

### Single-trial analysis of DA dynamics in NAc during motor-learning behavior

**Figure 5.**
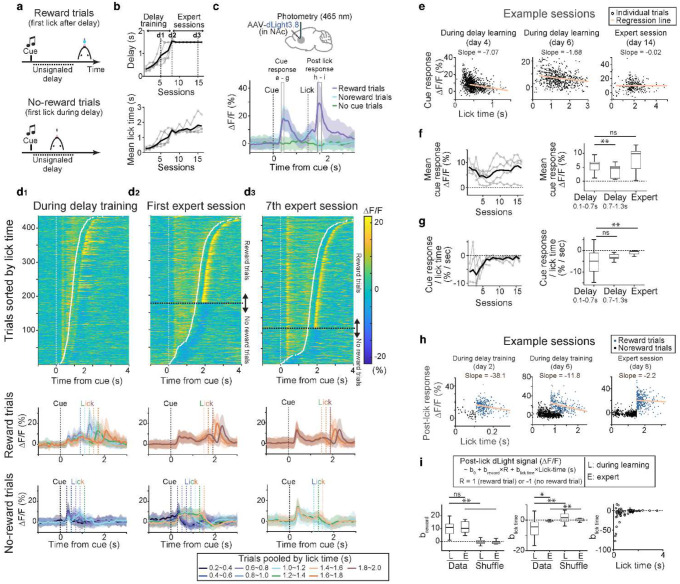
Fiber-photometry recording of DA dynamics across a motor learning task in mice. **a,** Schematic of a motor timing task. **b,** Learning curves for all animals with NAc photometry recording (n=4) across delay training and expert sessions. (Top) Delay duration. (Bottom) Mean time to first lick. Thin lines, individual animals, thick lines, average across animals. **c,** (Top) Schematic for recording NAc DA dynamics with fiber photometry. (Bottom) Average dLight responses aligned to trial onset in rewarded (blue), unrewarded (black), and nocue trials (green) in an example session with 1.5s fixed delay duration. Vertical dashed lines indicate timing of cue onset and lick. Magenta boxes indicate time windows for analysis of post-cue and post-lick DA responses. Responses are normalized to pre-cue activity. Shaded regions, bootstrap SEM. **d,** DA responses aligned to trial onset in three example sessions at different phases of learning. d_1_, day 5 of delay training. d_2_ and d_3_, days 1 and 6 of expert session with 1.5s fixed delay, respectively. Top row, rewarded trials. Middle row, unrewarded trials. Individual colors indicate the average ΔF/F of trials with different lick times. Lick times are indicated by vertical dotted lines. Shaded region, SEM. Bottom row, heat map of DA responses in all trials sorted by lick time (white dots). **e,** Relationship between first lick time and single-trial post-cue NAc DA response (0.3 ~0.4 s after cue onset) in 3 example sessions for rewarded (blue) and unrewarded (black) trials. Each dot represents individual trials. Magenta line, least squares fit. Slope, slope of the least square fit line. **f,** Left, mean post-cue NAc DA response per session. Thin lines, individual animals, thick lines average across animals. Right, average post-cue NAc DA response in sessions grouped by early delay training (delay duration 0.1~0.7s), late delay training (delay duration 0.7~1.3), and expert (1.5s fixed delay). Cue response shows a U-shaped curve, decreasing during delay training and increasing again in expert mice. Box plot center line shows the mean; box limits show upper and lower quartiles. **g,** (Left) Least squares regression slope of single-trial post-cue NAc DA response on lick time per session. Thin lines, individual animals, thick lines average across animals. (Right) Regression slope in sessions grouped as in (**f**). Cue response becomes less predictive of upcoming lick timing as animals learn to lick later. Box plot center line shows the mean; box limits show upper and lower quartiles. **h,** Relationship between first lick time and single-trial post-lick DA response (0.35~0.5 s after first lick) in 3 example sessions for rewarded (blue) and unrewarded (black) trials. Each dot represents individual trials. Magenta line, least squares fit for rewarded trials. Slope, slope of the least square fit line. **i,** Least squares regression of single-trial post-lick DA response on first lick time and reward outcome in learning (L) and expert (E) sessions. (Top) Regression model. (Bottom) Regression coefficients for reward (left) and lick time (middle), and the relation between regression coefficient for lick time and mean lick time in individual training sessions (right). Shuffle indicates regression with DA responses shuffled across trials. For the regression analysis, only lick time in the rewarded trials was considered, as the no-rewarded response was less modulated by lick time. * p<0.05, ** p<0.005, bootstrap. Box plot center line shows the mean; box limits show upper and lower quartiles.

Having established the high sensitivity of dLight3.8 across species and behavioral contexts, we finally asked whether dLight3.8’s enhanced sensitivity could capture rapid, trial-by-trial DA dynamics across learning. Given the critical role of DA in the moment-to-moment regulation of action, achieving single-trial resolution is essential for understanding how DA release shapes behavior^79^. We therefore recorded DA release in the NAc of head-fixed mice performing an operant motor learning task. In this task, head-fixed, water-restricted mice gradually learned to time their licking to obtain a water reward (Fig. 5a)^80^. Each trial begins with an auditory cue (3 kHz tone, 0.6s), followed by an unsignaled delay epoch. An early lick during the delay epoch aborts the trial without a reward (no-reward trials), whereas a lick after the delay epoch is rewarded (reward trials). For training, we gradually increased the delay duration from 0.1s to 1.8s based on the animal’s performance. Once the animal reached a 1.8 s delay, the delay duration was fixed at 1.5s (‘expert’). Following this protocol, mice learned to delay lick timing within and across sessions (Fig. 5b).

We conducted fiber photometry over 9–22 days, encompassing both training and the expert phase (Fig. 5c,d). Averaged fluorescence waveforms showed distinct patterns across reward, no-reward, and no-cue trials (Fig. 5c). In both reward and no-reward trials, a transient DA signal was detected following the cue, followed by a brief rise that peaked at the onset of licking. In reward trials, fluorescence intensity continued to rise to a large post-lick peak, whereas in no-reward trials, fluorescence decreased after the lick.

To further examine changes in DA dynamics across learning, we analyzed single-trial fluorescence responses of rewarded and non-rewarded trials across three key time points: delayed training, first expert session, and late expert session (Fig. 5d, **upper panel**). Averaged fluorescence waveforms across reward, no-reward during learning are also shown (Fig. 5d, **bottom panel**). The temporal dynamics of DA signals evolve across trials and sessions (Fig. 5e, **Extended Data Fig. 9d,e,i,j**). Within the session, the peak amplitude of the fluorescence response occurred shortly after the cue (i.e., cue-response) on individual trials and lick amplitude decreased with longer lick latencies during the early stage of delayed training (Fig. 5e). Across single trials during learning, the cue-response showed a U-shaped trajectory: high during initial cue association, decreasing as delay learning progressed, and re-emerging in expert phase (Fig. 5f, **Extended Data Fig. 9e,f**). To further explore whether the nature of DA coding differed across learning phases, we performed single-trial regression analysis of the cue response against lick timing. During early training, the cue response reliably predicted lick timing, indicating a movement-related signal; in contrast, in expert animals, cue responses were less predictive of lick timing, consistent with a transition to reward-related encoding, suggesting a shift in DA signaling from movement prediction to reward anticipation (Fig. 5g).

We also observed a robust DA transient after rewarded licks or a decrease in DA after unrewarded licks (Fig. 5c,d)^5,9,81^. The peak amplitude of post-lick fluorescence response (e.g. reward-response) was present throughout the different phases of training (Fig. 5d, **Extended Data Fig 9i-l**). Single-trial regression analysis of the reward response revealed that the post-lick DA response is strongly modulated by the lick timing in rewarded trials; a later rewarded lick resulted in a smaller post-lick DA response in the NAc (Fig. 5h), consistent with temporal discounting of DA responses with delayed rewards^82^. The modulation of post-lick DA responses by lick timing was more prominent during learning compared to expert sessions (Fig. 5i).

To examine if the dynamic change of DA release is region-specific, we also performed chronic recordings of DA signaling in the DLS across learning. In contrast to the NAc, cue-evoked responses were absent in the DLS during delayed training (**Extended Data Fig. 9q-x**). Although post-lick fluorescence signals were consistently observed throughout all training phases, these signals were less modulated by lick timing in the DLS.

The distinct DA dynamics observed in the DLS versus the NAc reflect region-specific contributions of striatal subcircuits during learning. This pattern aligns with prior electrophysiological recordings of midbrain DA neurons, which show early movement-related activity followed by the gradual emergence of reward-predictive cue responses^10,11^. The single-trial sensitivity of dLight3.8 now enables fiber photometry to capture these dynamic changes and subtle differences in targeted brain regions.

### Simultaneous fluorescence intensity and lifetime recordings

Though fiber-photometry recording of dLight3.8 permits detection of rapid, large DA transients triggered by specific tasks around brain regions with high fidelity, we have also observed slow, stochastic DA transients via two-photon imaging, suggesting potential tonic release (Fig. 2h and Fig. 3m,n). To achieve a quantitative measurement of DA release, it is ideal to combine intensity fluorescence changes, which capture the timing of transient DA dynamics, with fluorescence lifetime, which can be used to approximate DA concentration levels. dLight3.8 displayed large lifetime changes in HEK cells (Fig. 1d) and in acute brain slices (**Extended Data Fig. 10a-c**), we thus sought to ask if dLight3.8 is sensitive enough to detect behaviorally relevant DA release using fluorescence intensity and lifetime. We injected AAV-CAG-dLight3.8 into the NAc and recorded simultaneous intensity and lifetime changes using FLIPR during reward delivery (**Extended Data Fig. 10d**)^51^. Mice trained to collect chocolate pellets showed a robust fluorescence increase (ΔF/F = 64.5±22.9%, S.D.) and lifetime shift (ΔT=59.4±16.7 ps, S.D.) upon reward approach, lasting ~2 seconds (**Extended Data Fig. 10e,f**). These proof-of-concept results demonstrate that dual-mode dLight3.8 recordings can resolve both the timing and concentration of DA signals, making quantitative measurement of *in vivo* DA release possible.

## Discussion

To address the limitations of previous sensors in probing complex DA release dynamics that are highly variable in concentrations across brain regions *in vivo*, we developed and systematically characterized dLight3, a new generation of genetically encoded DA sensors built on the dLight1.3b scaffold and engineered to enhance both sensitivity and dynamic range.

Both novel variants (dLight3.6 and dLight3.8) achieved substantial improvements in signal-to-noise ratio and apparent affinity that permit robust measurements of broad-spectrum DA release beyond the striatum where transient or subtle DA release events are easily missed by prior sensors (Fig. 1c)^39,41^. dLight3.6 exhibited higher affinity for DA, making it comparable to dLight3.8 in detecting low-concentration signals, such as those observed in the SC and PFC during aversive stimuli or reward (Fig. 2f–i, Fig. 4, **Extended Data Fig. 7**).

dLight3.8 displays the largest dynamic range among all available DA sensors while also displaying DA-modulated lifetime changes. Notably, dLight3.8 was uniquely capable of recapitulating voltammetry waveforms showing high-amplitude DA transients modulated by DAT blocker (Fig. 1j, **Extended Data Fig. 3c-e**). dLight3.8 permits robust measurement of both optogenetically and behavior triggered release in rodents and flies (Fig. 3, **Extended Data Fig. 5**). The large dynamic range revealed dopamine fluctuations that span up to two orders of magnitude across different brain regions and behavioral tasks, representing a substantial improvement over recordings with the previous sensor (Fig.4). Single-trial analysis of DA transients revealed reward-history-dependent modulation and dynamic shifts in DA encoding during the progress of motor learning (Fig. 5, **Extended Data Fig. 9**).

Pharmacological manipulation of DA transport and signaling, such as through DA transporter (DAT) inhibitors, has long been used as a therapeutic strategy for neurological and psychiatric disorders^83,84^. Gaining a mechanistic understanding of how such manipulation alters both baseline DA levels and evoked release is therefore essential. The fluorescence response of single-FP based sensors is inherently dependent on basal ligand concentration and sensor dynamic range. Thus, limited dynamic range can result in a ceiling effect, masking further changes in DA levels, as observed with earlier dLight variants (**Extended Data Fig. 3c**)^34,39,85^. The expanded dynamic range of dLight3.8 overcomes this limitation, enabling accurate measurement of DAT inhibitor–induced increases in DA comparable to those captured by FSCV (Fig. 1j, **Extended Data Fig. 3d,e**). These results suggest that under conditions of DAT blockade, increased DA diffusion may enhance FSCV sampling but also likely contribute to increased electrically evoked release, as revealed by dLight3.8.

The expression level and pattern critically influence sensors’ performance. In addition to widely used promoters such as CAG and synapsin, we found that a dual-promoter tTA/TRE system with tTA expression in neurons and TRE-driven expression of dLight can significantly enhance basal brightness (**Extended Data Fig. 3a**)^86,87^. In addition, we observed that peak response amplitude strongly depends on expression patterns using Cre-driver line and viral delivery routes. When expressed in postsynaptic neurons (e.g., DRD1 cells), dLight3.6 and dLight3.8 produced DA responses over 20-fold greater than dLight1.3b (Fig. 1g,h). However, when expressed in presynaptic terminals (e.g., using DAT-Cre), the DA-induced amplitude changes of dLight3.6 and dLight3.8 decreased, while that of dLight1.3b increased, resulting in no significant amplitude differences among the three sensors in this context (Fig. 1h). These findings suggest that sensors with high affinity and large dynamic range should be targeted to postsynaptic cells to optimize detection of extracellular DA. Lower-affinity sensors like dLight1.3b may be advantageous for detecting higher DA concentrations at presynaptic terminals but are limited by their lower dynamic range at postsynaptic sites. It’s also possible that reuptake is slower and more spatially diffuse than release^88,89^. Thus, there is still room to improve the combined properties of dynamic range and affinity in the dLight3.0 series to enhance performance in presynaptic compartments. Similarly, systemic AAV delivery using the optimized capsid CAP-B10 led to higher SNR in striatum compared to PHP.eB and broader expression in PFC and HPC that wasn’t seen using PHP.eB (Fig. 2d,e, **Extended Data Fig. 4b**). Moving forward, a biophysical model of DA release, diffusion, and reuptake, paired with precise quantification of sensor expression levels in acute brain slices, will be essential for guiding sensor engineering to reach optimal intrinsic properties, such as apparent affinity and dynamic range.

Extending DA imaging beyond the striatum offers critical insight into the broader roles of DA in sensory and attentional processes. We therefore examined dLight3 performance in the SC, a region where earlier sensor versions failed to detect DA signals (Fig. 2f,h,i). The SC receives dopaminergic input from the zona incerta and locus coeruleus, with D1-type receptors enriched in the superficial SC (sSC), where DA inhibits neuronal activity^62,63,65^. *In vivo* two-photon imaging with dLight3.6 or dLight3.8 revealed spatially heterogeneous DA release, demonstrating the effectiveness of the novel sensors in detecting stimulus-evoked DA dynamics in brain regions sparsely innervated by DA inputs (Fig. 2h,i). DA dynamics were not detected with dLight1.3b, likely due to low sensitivity, though poor expression may also be a factor. The ability to study DA dynamics with dLight3 to salient stimuli in sSC revealed that looming visual stimuli evoked rapid decreases in fluorescence, suggesting transient DA suppression in sSC, while tail shocks induced sustained DA elevation lasting tens of seconds (Fig. 2h,i). These dynamics imply that looming stimuli may disinhibit sSC visual processing, whereas prolonged DA elevation following shock may bias processing toward deeper somatosensory pathways. Overall, DA responses to aversive stimuli highlight a broader role in modulating sensory salience and attentional gating.

We next benchmarked dLight3.0 against the GRAB-DA family of widely used GPCR-based DA sensors (GRAB-DA2m based on DRD2 and GRAB-DA3m based on DRD1) using optogenetic stimulation in both mice and flies. Optogenetic activation provides precise temporal control of dopaminergic neuron firing, enabling systematic comparison of sensor responses under defined stimulation parameters. In mice, dLight3.6 and dLight3.8 exhibited significantly larger fluorescence changes, 2 to 3-fold greater than GRAB-DA3 in the NAc following VTA stimulation, with dLight3.8 showing the highest sensitivity (Fig. 3a,c–f). In flies, dLight3.8 responses scaled with increasing stimulation frequency, whereas GRAB-DA2 signals saturated at the lowest frequency tested (Fig. 3b,g–i). These results highlight the superior sensitivity and dynamic range of dLight3.8 across species.

Next, we benchmarked the performance of dLight3.6 and dlight3.8 variants during behavior using simultaneous dual-fiber recordings from the NAc and PFC. Dopaminergic neurons in the VTA project to both the NAc and PFC, where they contribute to reinforcement learning, salience detection, and emotional regulation^1,3–7,30^. This approach allowed direct, within-subject comparison of DA dynamics across functionally distinct brain regions during reward and aversive learning. Across both foraging and fear-learning tasks, dLight3.8 exhibited the highest signal amplitudes, with up to a 14-fold improvement over dLight1.3b in the NAc and up to a 6-fold increase in the PFC (Fig. 4a–g). Historically, detecting reward-evoked DA responses in the PFC has been challenging^43^. However, the enhanced sensitivity of both dLight3.6 and dLight3.8 enabled robust detection of DA signals in response to both reward and shock in this region. Notably, shock-evoked signals in the PFC were significantly larger than reward responses, suggesting a heightened sensitivity to aversive salience. In addition, while dLight 3.8 outperformed dLight 3.6 in the NAc, the performance of the two variants was similar in the PFC (Fig 4a–g). This highlights the complementarity but distinct strengths of the two variants: dLight3.8 is superior for capturing the robust reward responses in the NAc, whereas the high affinity and faster dLight3.6 can equally resolve robust reward and aversive responses in the PFC. These dual-site recordings reveal that DA signaling during behavior is not uniformly distributed but instead reflects region-specific encoding strategies. These findings highlight the utility of high-sensitivity sensors and the importance of cross-region measurements to uncover the nuanced roles of neuromodulators in shaping complex behaviors.

Building on these region-specific findings during behavior, we next asked whether dLight3.8’s enhanced sensitivity could resolve DA dynamics at the single-trial level, a critical requirement for understanding how dynamic changes in DA release shape behavior across learning. We used dLight3.8 to track the dynamics of DA activity in the NAc throughout a complete learning paradigm (Fig. 5). Single-trial analysis revealed task-modulated, dynamic DA responses, with reward prediction error (RPE) signals emerging only in the later stages of learning, consistent with prior VTA electrical recordings^10^. Interestingly, DA responses to cues in the NAc did not encode upcoming movement timing, contrasting with previous findings in the dorsolateral striatum (DLS), where cue-evoked DA signals were predictive of movement timing (**Extended Data Fig. 9**)^1,2^. This discrepancy may reflect subregion-specific differences in DA responses to reward-predictive cues^15^. Instead, we observed a gradual increase in cue responses over days of training, consistent with region-specific differences in DA response timescales (Fig. 5d,g). Simultaneous recordings of DA release across multiple brain regions, combined with targeted manipulation of dopaminergic neurons, will be essential to further elucidate regional differences in DA signaling and their functional implications.

In summary, systematic benchmarking highlights the significantly enhanced performance of the dLight3 sensor series in capturing DA transients across a wide spectrum of amplitudes, brain regions, and behaviors that have historically challenged existing sensors. These experiments also demonstrated complementary yet distinct properties of both sensor variants, providing guidance for sensor selection tailored to specific experimental needs. Additionally, dLight3.8 supports both fluorescence intensity and fluorescence lifetime imaging (**Extended Data Fig. 10**). Because fluorescence lifetime is relatively insensitive to confounds such as sensor expression level, fiber placement, photobleaching, and hemodynamic interference, lifetime imaging offers a quantitative measurement of DA fluctuations and enables cross-region and brain state comparisons. Future development and integration of this approach may allow absolute quantification of extracellular DA concentrations, potentially resolving longstanding challenges in distinguishing tonic versus phasic DA release. Moreover, by combining lifetime-based detection with emerging technologies in real-time optical readout and closed-loop perturbation, the dLight3 platform opens the door to new paradigms for causal, quantitative interrogation of dopaminergic signaling across the brain.

## METHODS

### Reagent and key resources

All reagent, software and equipment are summarized in Supplementary Table 1.

#### Sensor Development and in vitro Characterization

##### General molecular biology

All DNA oligonucleotides were synthesized by Integrated DNA Technologies (Table SX). High-fidelity polymerase chain reactions (PCRs) were performed with Platinum SuperFi II Green PCR Master Mix (Invitrogen) according to the manufacturer’s instructions. PCR products were purified by column purification with buffers from QIAquick PCR Purification Kit (Qiagen) and columns from Syd Labs, Inc. Restriction enzymes, including DpnI, were sourced from New England Biolabs and used according to the manufacturer’s recommended protocols. All constructs were assembled via Gibson assembly17 using NEBuilder HiFi DNA Assembly Master Mix (New England Biolabs). Non-library plasmid DNA extractions were performed with QIAprep Spin Miniprep Kit buffers (Qiagen) and columns from Syd Labs, Inc. pAAV-hSyn-GRAB_ACh3.0 was a gift from Yulong Li (Addgene plasmid # 121922; http://n2t.net/addgene:121922; RRID:Addgene_121922)5. DH10β bacteria were purchased from Invitrogen and electrocompetent cells were prepared in house. All Sanger sequencing reactions were performed by Genewiz from Azenta Life Sciences. HEK293T cells (CRL-3216) were obtained from the American Type Culture Collection.

##### Plasmid construction for engineering

To generate the starting construct for the de novo engineering approach, the sequence encoding for cpEGFP in dLight1.3b was replaced with the cpEGFP from GRABACh 3.0 (ref. 5) via assembly. The vector backbone with just the DRD1 receptor of dLight1.3b was amplified to generate a linear vector fragment without the cpEGFP and the insert fragment encoding cpEGFP from GRABACh 3.0 with homologous 5’ and 3’ sequences with the linearized vector was generated using oligonucleotides carrying the homologous sequences to enable plasmid assembly. For ease of use and to facilitate engineering, constructs were also cloned from the original pCMV backbone (pCMV; ref. 3) in the early stages of engineering to a modified pMiniDisplay backbone (pMDm; ref. 18), with the region encoding from the immunoglobulin signal peptide to the platelet derived growth factor receptor (PDGR) replaced with the constructs of interest, via assembly. Assembly reactions were then used to transform Escherichia coli.

All transformations were performed with electrocompetent DH10β E. coli (Invitrogen). Transformed bacteria were plated on LB (10 g/mL NaCl, Miller formulation) agar plates supplemented with either 400 μg/mL ampicillin (Thermo Fisher Scientific) for pMDm-based constructs or 50 μg/mL kanamycin (Thermo Fisher Scientific) for pCMV constructs and grown overnight at 37 °C. Plasmids were extracted from individual colonies grown in 3 mL liquid 2YT cultures supplemented with the appropriate antibiotics and confirmed by Sanger sequencing.

##### Library creation

Site-directed mutagenesis libraries for a single position were generated by high-fidelity PCR amplification and forward and reverse primers carrying the codons for desired mutations followed by DpnI digestion and an assembly reaction for circularization to improve transformation efficiency prior to transformation of E. coli. The preparation of site-directed mutagenesis libraries for two positions was similar to our general cloning strategy using Gibson assembly17 though the forward and reverse primers of the insert carried the codons for the desired mutations in addition to the homologous sequences with the vector. All site-directed mutagenesis libraries were generated using the 22-codon trick19 to minimize library size. In all cases, DH10β E. coli were transformed with the assembly reactions via electroporation prior to plating on LB agar plates. E. coli was also separately transformed with the starting template used for each library and plated.

Individual colonies from the library, as well as at least four individual colonies from the starting template plate, were cultured in 800 μL of 2YT media supplemented with antibiotics in 2.2 mL 96-well deepwell plates (Thermo Scientific) overnight at either 30 °C or 37 °C. Plasmids from these libraries were then extracted with an Opentrons OT-2 liquid handling system (Opentrons) using the Wizard^®^ MagneSil^®^ Plasmid Purification System (Promega). After extraction, an average DNA concentration for each plate was obtained by averaging the concentrations of 12 to 16 wells as measured on a NanoDrop^™^ 2000 (Thermo Scientific).

##### *in vitro* screening

Libraries were screened in HEK293T cells transfected with Lipofectamine 2000 (Invitrogen) via reverse transfection. Briefly, 96-well glass-bottom plates were coated with poly-D-lysine for at least 1 hour, washed at least three times with ddH2O and dried before use. Transfection complexes with 0.225 μL of Lipofectamine 2000 and an average of 75 ng of plasmid DNA per well were prepared by the Opentrons OT-2 liquid handling system. HEK293T cells (~ 25,000 cells per well) were added on top of the transfection complexes and incubated at 37 °C with 5 % CO2 for 2 days before imaging. HEK293T cells were maintained in Dulbecco’s Modified Eagle’s Medium (DMEM; Gibco) supplemented with 10% fetal bovine serum (FBS; Gibco) and 1% streptomycin/penicillin (Gibco).

Cells were imaged in Hanks’ Balanced Salt Solution (HBSS; Gibco) supplemented with 10 mM HEPES (pH = 7.4) and washed with the same at least twice prior to imaging. Images were acquired by an ImageXpress Micro Confocal High-Content Imaging system (Molecular Devices) equipped with a 20 × air objective and 475/34 nm excitation and 536/40 nm emission filters in widefield mode. Each well was imaged under three conditions (apo, 500 nM or 1 μM, and 150 μM or 200 μM final [DA]) by successive addition of concentrated DA solutions prepared prior to imaging by dissolving DA in the imaging buffer. Images were analyzed offline with custom Matlab scripts to calculate F0 and ΔF/F0 for both DA concentrations.

E. coli was transformed with plasmids encoding promising variants for plasmid propagation and the DNA used for transfecting HEK293T cells in 24-well plates (~120,000 cells per well) for further validation on an inverted Zeiss Observer LSN710 confocal microscope using a 40× oil-based objective, a 488 nm laser for excitation, and collecting the emission from 493 to 598 nm or on an inverted Leica DMi8 microscope using a 40× or 63× oil-based objective, a 488 nm OPSL with emission between 495 to 750 nm collected. Plasmids for winning variants were then sequenced by Sanger sequencing and used as the templates for the next round of engineering with separate libraries prepared for each template in the case of multiple winning variants in a round. Stable cell lines for dLight1.3b, dLight3.6 and dLight3.8 were generated using Flip-In T-Rex 293 cells (Thermo Fisher Scientific), according to the manufacturer’s protocol.

##### Viral preparations

cDNAs encoding the dLight dopamine sensor variants (Figure 2B) were cloned into AAV plasmids with human Synapsin1 (hSyn1) promoter (Addgene#111068), CAG promotor (Addgene#59462), Flex-CAG promoter (Addgene#99280) and TRE promoter (Addgene#104110). tTA/TRE-dLight single vector was generated based on the AAV-SynTetOff vector20 using tTA plasmid (Addgene #104109) and TRE plasmid (Addgene #104111). The inducible hSyn1 (ihSyn) and the minWPRE fragments21,22 were used. The hSyn-tTA/TRE-dLight constructs were also prepared as Cre and non-Cre dependent versions.

Production and purification of AAV vectors were performed as follows. HEK293T cells were seeded at 4–6 million cells in two 100 mm plates in Dulbecco’s modified Eagle’s medium containing 2 % fetal bovine serum (FBS) 24h prior to the transfection. AAV serotype, Helper, and expression plasmids were co-transfected by calcium phosphate transfection method. Cells were washed with DPBS 6h after transfection and further incubated with the medium containing 2% FBS. Cells containing virus particles were collected 60~84 h after medium replacement. After extraction by four cycles of freeze-and-thaw, the virus particles were purified from the crude lysate by three rounds of centrifugation. For hSyn-tTA/TRE-dLight single virus, the viruses were purified using chloroform-based extraction method23.

##### Generating transgenic *Drosophila* lines:

dLight3.8 sequence from above mentioned AAV plasmid was subcloned in place of mCD8-GFP sequence in pJFRC7–20XUAS-IVS-mCD8::GFP plasmid (Addgene # 26220). Stable transgenic *Drosophila* lines (UAS-dLight3.8) were generated by site-directed Phi-C31 based insertion into 260B landing site (VDRC#60100). Injections were performed by BestGene Inc. Multiple transformants were tested and the best one was chosen for experiments. Stable lines including insertions at other landing sites, will be deposited at Bloomington Drosophila Stock Center (BDSC) for sharing with the community (also available on request from Bidaye lab).

#### *Ex vivo* Characterization

##### Viral transduction in mice for 2p slice experiments

AAVs encoding dLight variants were injected into C57Bl6/J, DAT-Cre, and D1-Cre mice (postnatal weeks 8–10) under isoflurane anesthesia using a pulled glass micropipette (Narishige PE-22) mounted on a Nanoject III (Drummond). Mice were placed in a stereotaxic frame (David Kopf Instruments), and injections were performed under aseptic conditions. For dorsal striatum targeting, 400–800 nl of virus was injected at AP +0.9 mm, ML −2.0 mm, DV −2.2 mm. For SNc injections in DAT-Cre mice, coordinates were AP −3.15 mm, ML +1.25 mm, DV −4.15 mm. For prefrontal cortex injections (Cg2/A24a), 200 nl was injected at AP +1.3 mm, ML +0.4 mm, DV −1.68 mm. All DV values were measured from the cortical surface. Injections were delivered at 1–2 nl s^−1^, and the pipette was left in place for 5 min post-injection, then withdrawn gradually. Mice received carprofen (5 mg kg^−1^, s.c.) at the end of surgery and post-operative analgesia via medicated gel for 72 h. Animals were used ≥3 weeks post-injection.

##### Acute brain slices preparation

Three to four weeks after viral injection, adult mice were anesthetized with isoflurane and either decapitated (for electrical stimulation experiments) or transcardially perfused with ice-cold, carbogenated ACSF (for FLIM experiments). For stimulation experiments, brains were rapidly extracted and chilled in cutting aCSF containing 92 mM NMDG, 2.5 mM KCl, 1.25 mM NaH2PO4, 30 mM NaHCO3, 20 mM HEPES, 25 mM glucose, 2 mM thiourea, 5 mM sodium ascorbate, 3 mM sodium pyruvate, 0.5 mM CaCl2, and 10 mM MgCl2 (pH 7.38–7.4, ~310 mOsm), bubbled with 95% O2/5% CO2. Brains were blocked and sliced (300 μm) in an oblique plane (45° rostral-up) using a vibratome (VT1000S, Leica) to preserve corticostriatal projections. Slices recovered for ≤12 min at 34 °C in oxygenated cutting aCSF, then transferred to holding aCSF (92 mM NaCl, 2.5 mM KCl, 1.25 mM NaH2PO4, 30 mM NaHCO3, 20 mM HEPES, 25 mM glucose, 2 mM thiourea, 5 mM sodium ascorbate, 3 mM sodium pyruvate, 2 mM CaCl2, 2 mM MgCl2; pH 7.35–7.4, ~300 mOsm) at room temperature until use. For FLIM experiments, brains were placed in ice-cold, carbogenated ACSF containing 125 mM NaCl, 2.5 mM KCl, 25 mM NaHCO3, 2 mM CaCl2, 1 mM MgCl2, 1.25 mM NaH2PO4, and 17 mM glucose (300 mOsm kg^−1^). Slices (300 μm) were prepared in ice-cold ACSF and incubated for 10 min at 34 °C in choline-based solution containing 110 mM choline chloride, 25 mM NaHCO3, 2.5 mM KCl, 7 mM MgCl2, 0.5 mM CaCl2, 1.25 mM NaH2PO4, 25 mM glucose, 11.6 mM sodium ascorbate, and 3.1 mM sodium pyruvate (320 mOsm kg^−1^). Slices were then transferred to oxygenated ACSF at 34 °C for 1 h before being held at room temperature. For FLIM recordings, slices were maintained at 32–34 °C.

##### Fast-scan cyclic voltammetry and Electrical Stimulation

After recovery, slices were transferred to a recording chamber perfused with oxygenated ACSF (119 mM NaCl, 2.5 mM KCl, 1.25 mM NaH2PO4, 24 mM NaHCO3, 12.5 mM glucose, 2 mM CaCl2, 2 mM MgCl2; pH 7.35–7.4, 295–300 mOsm) maintained at 36.5 °C. Phasic dopamine release in the dorsal striatum was evoked by 2 s, 10 Hz trains (2 ms pulse width, 1–5 V) delivered every 5 min via a bipolar stimulating electrode (two SNEX-100 PI concentric electrodes epoxied side-by-side, MicroProbes), triggered by a custom-built stimulator. In PFC slices, stimulation consisted of a pseudo-one-pulse (p1P) protocol comprising four biphasic pulses (2 ms, 60 Hz within 30 ms) to minimize autoreceptor-mediated inhibition.

Dopamine release was measured using fast-scan cyclic voltammetry (FSCV) with a 10 μm-diameter carbon fiber microelectrode (CF10–100 or CF10–250, WPI) positioned <0.5 mm from the stimulation site. A triangular waveform (−0.4 V to +1.3 V to −0.4 V, 8.5 ms) was applied at 10 Hz using a CV-7B headstage and Multiclamp 700B amplifier (Molecular Devices), and data were acquired using pCLAMP 10.7 software. Electrodes were calibrated in 0.2–1 μM dopamine standards (Sigma) prepared in PBS with 0.1 M perchloric acid using a reference electrode (RE-1B, ALS Co., Japan). Following three stable baseline responses, drug-containing ACSF (e.g., methylphenidate, 30 μM) was bath-applied for ≥15 min before resuming stimulation.

FSCV data were initially analyzed using custom scripts in AxoGraph X (v1.7.6) and subsequently processed using an automated Python-based pipeline. Voltage-clamp data were extracted from ABF files using the pyABF package (Harden, 2020). Dopamine oxidation currents were averaged over the +0.45 V to +0.55 V window and converted to concentration (nM) using the in vitro calibration factor. Dopamine transients were aligned to stimulation onset and plotted relative to baseline, defined as the mean signal from 0.90 to 0.95 s prior to stimulus.

##### Acute slice imaging

Epifluorescence imaging of dLight signals was performed using a BX51WI microscope (Olympus) equipped with a GFP filter set (U-MWIB3, Ex BP460–495, DM505, Em BA510IF) and a stable white light source with liquid light guide (U-HGLGPS, Olympus). Images (2560 × 2160 pixels, 16-bit) were acquired at 10 Hz using a sCMOS camera (pco.edge 5.5 mono) and CamWare software, with shutter control (VMM-T1, Uniblitz) and acquisition triggered by pCLAMP. Imaging and stimulation protocols were computer-controlled to ensure reproducibility and minimize disturbance to the sample.

Each imaging session consisted of 194 frames acquired at 100 ms intervals. A 200 × 200 μm region of interest (ROI), positioned near but not directly adjacent to the stimulating electrode, was used to compute average fluorescence intensity over time, F(t). Prior to stimulation, F(t) exhibited a biphasic exponential decay modeled as F₀(t) = A·exp(−t/τ₁) + B·exp(−t/τ₂) + C. This baseline curve, fitted individually for each sequence, was extrapolated to post-stimulus timepoints to calculate ΔF/F₀ = (F − F₀)/F₀. This approach emphasized early post-stimulus dynamics but was not used to quantify fluorescence decay.

Two-photon (2P) imaging data were analyzed with custom MATLAB scripts. For each time series, mean fluorescence across the field of view (40× objective) was extracted over time. ΔF/F₀ was calculated as (F(t) − F₀)/F₀, with F₀ defined as the pre-stimulus baseline. Datasets with low expression (ΔF/F₀ < 0.25% at 0.3 s post-stimulus) were excluded from analysis. No photobleaching correction was applied due to stable signal at 33 Hz acquisition. Images in figures were processed using Imaris (v9.5.1) and Fiji (ImageJ). For time-series visualization (e.g., Fig. 2C), maximum intensity projections were performed over two frames at representative timepoints: baseline, first peak (immediately after stimulation), second peak or plateau (depending on dLight variant), and recovery (5 frames post-stimulation).

##### Fluorescence lifetime microscopy (FLiM)

Lifetime was measured with a custom-built 2-photon microscope in which fluorophores were excited at 920 nm using a Ti:Sapphire laser (Chameleon Vision II, 80 MHz, Coherent, Santa Clara, CA). Emission photons were collected with a fast photomultiplier tube (H7422–40MOD, Hamamatsu). Single photon counting data was acquired using a high-speed, 2.7 GHz vDAQ system (MBF Bioscience, Ashburn, VA) through ScanImage v2021.0.0 in MATLAB 2021b. Data was binned at 0.391ns for 31 bins per pulse starting from the reference laser pulse. Time bin 32 counted photons in the entire 12.5ns period to achieve real time fluorescence intensity visualization. For each field of view, data was collected at 10x zoom, 1.07 Hz, at a resolution of 512 × 512 pixels. Photons from 10 frames were collected sequentially and were pooled in a single image to an effective sampling rate ~0.1 Hz. For each field of view, the top 66% brightest pixels were taken as effective pixels, as in prior studies (Ma et al., 2024).

For representative images, the lifetime was calculated on a per pixel basis, and for field of view analysis, all pixels were pooled together. The average lifetime was determined using the following formula:

[Equation]

Where F(t) is the number of photons in a given 0.391 ns time bin, and t is the corresponding time bin. Three images were collected per field of view and the lifetime per image was calculated and averaged to determine the lifetime per field of view.

##### 2-photon *ex vivo* imaging in *Drosophila*

For functional validation of dLight3.8 in Drosophila, we optogenetically activated dopaminergic TH-Gal4 neurons while recording dLight3.8 or GRAB-DA2m signal from the same neurons. To this end, brains of flies (6–9 days old) of the genotypes (1) TH-Gal4>UAS-ChrimsonR::mCherry; UAS-dLight3.8, (2) TH-Gal4> +; dLight3.8 and (3) THGal4>UAS-ChrimsonR::mCherry; GRAB-DA2m were dissected in extracellular saline solution bubbled with carbogen and transferred on a poly-l-lysine-coated coverslip fixed in an imaging chamber (ALAMS-518SWPW). Imaging experiments were performed under a Bergamo II two-photon microscope using a ×20 objective lens (numerical aperture 1.0, XLUMPLFLN, Olympus). Throughout imaging, a perfusion system (78018–40, Masterflex) ensured the delivery and circulation of bubbled extracellular saline with carbogen onto the brains. Single-plane imaging of dLight3.8 or GRAB-DA2m signal was recorded at 15 Hz in ScanImage software (MBF Bioscience), using a 920 nm Ti:Sapphire laser (MaiTai DeepSee, Newport Spectra-Physics). For optogenetic stimulation, a fibre-coupled 655 nm LED (FC1-LED, Prizmatix) was positioned towards the brains with a micromanipulator (Misumi XYZFG2) to deliver 2 s red light (0.08 mW/mm2) at different frequencies (20, 33, 50, 100 Hz) in a random manner, with an inter-stimulation interval greater than 10 s. The LED was controlled and synchronized with the resonance imaging scanner (8.3 kHz) using ScanImage vDAQ, such that red light was delivered only during the non-imaging fly-back time of the scanner. Background subtracted imaging data were analyzed using ImageJ

#### *In vivo* Experiments

##### Animals

Mice were group housed (2–4 per cage) under standard laboratory conditions on a 12-h light/12-h dark cycle with ad libitum access to food and water. All procedures were approved by the Institutional Animal Care and Use Committees (IACUC) of the Okinawa Institute of Science and Technology, University of California Davis, University of California Berkeley, Max Planck Florida Institute, Allen Institute, and in accordance with NIH guidelines, Japanese law, and the Public Health Service Policy on Humane Care and Use of Laboratory Animals. All institutions are accredited by the Association for Assessment and Accreditation of Laboratory Animal Care International (AAALAC). Both male and female mice were used for all experiments. No sex differences were observed, and data were pooled across sexes. Animals were adults (>8 weeks old) at the time of experimentation. Transgenic mouse lines used included DAT-IRES-Cre (Jackson Laboratories, #006660), D1-Cre [Tg(Drd1-cre)FK150Gsat, MGI:3836633], and D1-Cre (MMRRC, #037156-JAX). Control wild-type C57Bl6/J mice were obtained from Charles River Japan and Envigo USA. Adult male Long-Evans rats were also used for select behavioral experiments in accordance with UC Davis IACUC protocols. Drosophila melanogaster were raised on standard medium at 25°C, 60% humidity in a 12/12 h light–dark cycle. For optogenetic experiments, experimental flies were collected on food supplemented with all-trans retinal (100mM final concentration; cat: R2500; CAS: 116–31-4, Sigma-Aldrich) and kept in darkness before testing.

##### Surgical Preparations

Adult mice (>8 weeks old) and male Long-Evans rats were anesthetized with isoflurane (induction: 3–4%; maintenance: 1–2%) and placed in a stereotaxic apparatus (Kopf Instruments). Additional analgesics, such as meloxicam (5–10 mg/kg, i.p.) or ketofen (5 mg/kg, i.p.), were administered perioperatively. For rats, ketamine (30 mg/kg, i.p.) and buprenorphine (0.02 mg/kg, i.p.) were also used at induction. Body temperature was maintained throughout surgery.

A small craniotomy was performed over the target site using a dental drill. AAVs encoding dLight variants (e.g., dLight1.3b, dLight3.6, dLight3.8) were injected using pulled glass pipettes (tip diameter 15–30 μm) or nanoliter injector systems (Nanoject III or Nanofil, Drummond or WPI), at rates of 2 nL/s, with injection volumes ranging from 20 nL to 800 nL depending on experiment and species. Injections targeted regions including the nucleus accumbens (NAc), dorsal and tail striatum, medial prefrontal cortex (mPFC), basolateral amygdala (BLA), ventral tegmental area (VTA), dorsal medial striatum (DMS), and superior colliculus (SC) (coordinates summarized in table below). Pipettes were held in place for several minutes post-injection to minimize backflow.

Following virus delivery, implants were positioned 100–200 μm above the injection site. These included optical fibers (200–400 μm core, 0.37–0.66 NA, Doric or Neurophotometrics), dual-ferrule assemblies of tapered fibers, or chronic cranial windows for two-photon imaging. Implants were secured with light-curing optical adhesive (Loctite 4305), dental acrylic (Lang Dental), or adhesive cement (RelyX Unicem 2, 3M). For the SC imaging experiment, the transverse sinus was ligated and transected to expose the underlying structure, and a custom cranial window was implanted with cyanoacrylate and optical adhesive and a custom titanium or stainless-steel headpost was affixed to the skull with Metabond (Parkell). Animals recovered for 2–3 weeks before behavioral training or in vivo imaging.

**Table:** 

Experiment	Brain Region	A/P	M/L	D/V
Fluorescence Lifetime Photometry	Nucleus Accumbens Core	+1.2 mm relative to bregma	±1.3 mm relative to bregma	4.1 mm below brain surface
	Tail of Striatum	−1.35 mm relative to bregma	±3.28mm relative to bregma	2.4 mm below brain surface
2-photon Imaging	Superior Colliculus	80 to 1300 μm rostral from the rostral edge of the transverse sinus	90 to 820 μm lateral from the left edge of the central sinus	70 – 300 μm below the surface of the SC
Optogenetics	Nucleus Accumbens	+1.0 mm relative to bregma	−1.35 mm relative to bregma	−4.1 mm below brain surface
	Basal Amygdala	−1.6 mm relative to bregma	−3.3 mm relative to bregma	−4.3 mm below brain surface
	Ventral Tegmental Area	−3.05 mm relative to bregma	−0.6 mm relative to bregma	−4.2 mm below brain surface
Dual-site mouse behavior	Prefrontal Cortex	+1.78 mm relative to bregma	±0.27mm relative to bregma	Inject at −2.75 and −2.25 mm, fiber tip at −3 mm relative to bregma
	Nucleus Accumbens	+1.45 mm relative to bregma	±1.5 mm relative to bregma	Inject at −4.75 and −4.25mm, fiber tip at −5 mm relative to bregma
Dual-site rat behavior	Prelimbic Cortex	+3.0 mm relative to bregma	±0.7 mm relative to bregma	Inject at −3.4 mm and −2.8 mm, fiber tip at −3.75 mm below brain surface
	Dorsal Medial Striatum	+1.2 mm relative to bregma	±2.2 mm relative to bregma	Inject at −4.0 mm and −3.4 mm, fiber tip at −4.35 mm below brain surface
Motor Timing Task	Nucleus Accumbens	+1.45 mm relative to bregma	−1.5 mm relative to bregma	−4.5 mm relative to bregma

#### *in vivo* Recordings

##### 2-photon Imaging in mice

Two-photon imaging was performed in head-fixed awake mice 4–17 weeks after surgery. Mice were habituated over 4 daily sessions prior to imaging. A home-built adaptive optics two-photon microscope equipped with a femtosecond laser (InSight DeepSee, Spectra-Physics) tuned to 940 nm was used for excitation. Imaging was performed with a 10× (0.6 NA, Olympus) or 16× (0.8 NA, Nikon) objective. Optical aberrations from the system and cranial window were corrected using adaptive optics and 2 μm red fluorescent beads applied during surgery. Images were acquired at 2.2 Hz (300 × 300 pixels, 0.5–2 μm/pixel) with a custom LabVIEW program. The imaging depth was 50–100 μm below the superior colliculus surface, estimated using the bead layer. Laser power ranged from 50–90 mW post-objective, and imaging sessions lasted 1–4.5 hours.

##### 2-photon Imaging in *Drosophila*

For functional connectivity validation of dLight3.8 in behaving flies, we presented a sugar reward to tethered fly walking on the ball while recording dLight3.8 signal in the mushroom bodies (MB), neuropils where several dopamine neurons encoding reward or punishments project to (Waddell 2013; Zolin et al 2021). In brief, female flies (7–9 days old) of the genotype OK107-Gal4>UAS-dLight3.8 were anaesthetized on ice and tethered on a custom fly holder. The head cuticle was removed and the brains were exposed for subsequent imaging. After dissection, the holder was placed under the two-photon microscope, and an air-supported ball was positioned under the flies. The temperature at the objective (30–35 °C) was maintained with a heater paired to the air–ball system and bubbled extracellular saline with carbogen was delivered throughout imaging using the perfusion system mentioned in the previous section. A volume containing the mushroom body vertical lobes was recorded at 920 nm using a fast z-piezo device, resulting in a volumetric rate of 12.5 Hz. While imaging, a 1 M sucrose droplet placed on small piece of aluminum foil was delivered to the flies using a micromanipulator, with an inter-presentation interval greater than 10 s. A given session typically consisted of 2–3 sucrose presentations. Fly movies (720 × 540 resolution; 200 Hz) were acquired with FLIR BlackFly-S camera and synchronized to dLight3.8 imaging using ScanImage vDAQ, as described in Sapkal et al 2024, Nature. The synchronized imaging and fly feeding behavior data were analyzed offline.

##### Fiber Photometry

Fiber photometry recordings were performed across multiple behavioral paradigms listed below using either commercial or custom-built systems. In all experiments, excitation light was delivered through low-autofluorescence patch cords (200–400 μm core, 0.37–0.66 NA) connected to implanted optical fibers via zirconia sleeves. Excitation was typically provided at 470/490 nm (dLight activation) and 405/430 nm (isosbestic control) using sinusoidal or pulsed LED modulation. Emission light (centered at 525 nm) was collected through the same fiber and either directed to femtowatt photodetectors (Newport) or CMOS cameras, depending on the system. LED power was calibrated to 35–50 μW at the fiber tip.

Fluorescence lifetime photometry (FLIPR) recordings were performed at 10 kHz using a time-correlated single-photon counting system. Mice were recorded during a pellet retrieval task, and fluorescence lifetime and intensity were downsampled to 10 Hz using a rolling mean and aligned to the time of reward consumption. Behavior and FLiP recordings were synchronized via Bonsai, and ΔF was calculated relative to the −10 to −5 s pre-consumption baseline.

For all photometry experiments, signals were motion-corrected and low-pass filtered. ΔF/F was calculated using the fitted isosbestic or baseline trend to correct for photobleaching and slow drift.

##### Optogenetic Stimulation

Optogenetic stimulation (e.g., VTA ChrimsonR) was performed through the implanted fiber using a 625–630 nm LED (2–5 mW at the tip), delivered either continuously or in pulse trains (e.g., 5–20 Hz, 15 ms pulses). Stimulation epochs were interleaved with imaging channels to avoid spectral overlap.

#### Behavior Testing

##### Visual stimulation and tail shock for superior colliculus recordings

Visual stimuli were generated with Psychophysics Toolbox and back-projected onto a Teflon-coated screen (13.5 cm from the right eye, 20° angled) using a modified DLP projector (Lightspeed DepthQ-WXGA-360). The stimulus field spanned 80° × 80° visual angle. Looming stimuli consisted of a black circle expanding from 1 pixel to 80° at 80°/s, following a 10 s full-screen illumination. Each looming trial (20 s) was interleaved with non-looming trials (40 total each/session). Tail shocks (100 μA–2 mA, 0.5 s) were delivered during separate trials via a precision shocker (Coulbourn Instruments) through electrode gel-coated wires affixed to the tail. Shock and non-shock trials (20 each/session) were 40 s long, with 10 s baseline and 29.5 s post-event. Each animal received 1–3 shock sessions total.

##### Reward Foraging Behavior and Fear conditioning

Behavior training was done in a custom behavior training setup. Mice were trained in a two-port reward foraging task. A tone signaled trial start, and mice were required to nose-poke the “action port” to arm the “reward port” for reward collection. Inter-trial intervals (ITIs) were 10–20 s. For fear conditioning, mice received 10 tone-only presentations (20 s, 3 kHz) on day 1, followed by 20 tone–shock pairings (0.3 mA, 1 s footshock) on day 2, and a repeat of day 1 on day 3. Trials were spaced at 2-min intervals.

##### Two-Armed Bandit Task (Rats)

Rats were trained to initiate trials via center-port nose-pokes, followed by choice between two side ports with different reward probabilities. Hold durations increased gradually from 0.1 to 1.5 s. After training with fixed 80%/20% reward contingencies (switching after 15–30 high-reward choices), rats progressed to pseudo-random reward blocks (20%, 40%, 80%, port identity counterbalanced) over several sessions.

##### Motor Timing Task

Mice were trained following published protocols (Majumder et al., 2023) in two phases: cue association (2–3 days) and delay training (~6 days). Trials began with a 3-kHz tone (3 repeats, 150 ms), followed by a delay epoch. Licking during the delay aborted the trial. Correct licks during the 3 s answer epoch yielded a 2 μL water reward. ITIs were drawn from an exponential distribution (mean: 2.2 s, min: 0.3 s). Cue-omission trials were included to assess spontaneous licking. Delay duration increased incrementally to 1.8 s based on performance. Expert animals were tested at a fixed 1.5 s delay.

#### Perfusion and slice histology

Brain tissue was collected by transcardial perfusion. Mice were anesthetized with 5% isoflurane and perfused intracardially with 10 mL of saline (0.9% NaCl) followed by 50mL of PFA 4% paraformaldehyde (PFA) at a flow rate of 9mL/min. Brains were then postfixed in 4% PFA at room temperature (22–25 °C) for 3–6 hours, and overnight at 4°C. Brains were stored in PBS with 30% sucrose. To evaluate fiber placement and virus expression, brains were sectioned coronally at 100 μm using a Microm HM400 Sliding Microtome. Sections were then incubated overnight at room temperature (RT) in blocking solution (5% Normal Goat Serum, 4M Urea, 0.2% Triton X-100). Sections were then incubated for 3 days at room temperature in primary antibody solution (anti-GFP, 1:1000, Abcam; catalog no. Ab13970, in blocking solution) and then washed with 1xPBS (six times for 10 min) and incubated for 1 day at room temperature in secondary antibody solution (Goat anti-Chicken Alexa Fluor^™^ 488, 1:500, Invitrogen; catalog no. A-11039, and 4’,6-diamidino-2-phenylindole, 1:1000, Invitrogen; catalog no. d1306, in blocking solution). Next, sections were washed with 1xPBS (six times for 10 min) and mounted using ProLong^™^ Diamond Antifade Mountant. Sections were imaged on an Olympus VS110 slide scanner, an Olympus FV3000 confocal microscope, or a Leica DMI8 Confocal Microscope.

*Drosophila* brains from adult flies (5–7 days old) of the genotype TH-Gal4>UAS-ChrimsonR::mCherry; UAS-dLight3.8 were dissected and immunostained as described Wu et al (2016) with detailed protocols available at (https://www.janelia.org/project-team/flylight/protocols). Primary antibodies used were chicken anti-GFP (1:1,000, Thermo Fisher Scientific, AB_2534023) to label dLight3.8, rabbit anti-dsRed (1:500, CloneTech, AB_10013483) to label mCherry, and anti-Bruchpilot (1:500, nc82, mouse monoclonal, Developmental Studies Hybridoma Bank, AB_2314866) to label brain neuropils. Alexa fluor secondary antibodies (Thermo Fisher Scientific) were used at 1:500 dilution (Goat antichicken, Alexa488, AB_2576217; Goat antirabbit, Alex568, AB_10563566; Goat antimouse, Alex568, AB_2534072 and Goat antimouse, Alex647, AB_141725). Images were obtained on Zeiss LSM780 confocal microscope and processed in ImageJ (version 1.8.0_172, Fiji ImageJ).

#### Data Analysis

##### *in vivo* 2-Photon Imaging Mouse Data

Two-photon imaging data were analyzed using ImageJ and custom MATLAB scripts. Motion correction was performed using an iterative cross-correlation-based rigid registration algorithm. In looming stimulus experiments, visual stimulation artifacts were corrected by subtracting frame-wise luminance changes estimated from control trials recorded with minimal laser power. ΔF/F₀ was computed by averaging fluorescence across all pixels in the field of view or within defined ROIs (3×3 grid of equal-sized squares), with F₀ defined from baseline frames preceding stimulus onset (7–2 frames before looming onset or final 5 frames before tail shock). Peak responses were calculated as the minimum ΔF/F₀ during 1.4–2.7 s post-stimulus for looming, and mean ΔF/F₀ during 3.2–5.5 s post-shock for tail shock. Differences between early and late trials were assessed using paired t-tests.

##### *Drosophila* 2-Photon Imaging Data:

*Ex vivo* imaging: In Fig. 3g, change in dLight3.8 or GRAB-DA2m signal from the dopaminergic TH-neurons projecting towards the mushroom body vertical lobe was calculated using ΔF/F = (F − F0)/F0, where F0 is the mean fluorescence intensity 2 s before stimulation. In Fig.3h the peak ΔF/F was calculated for each stimulation frequency. In Fig.3i, the fold change was computed as the ratio of peak ΔF/F responses of dLight3.8 to the average peak ΔF/F response of GRAB-DA2m at each stimulation frequency.

*In-vivo* imaging: In Fig. 3l, the averaged stimulus zΔF heatmap was generated using a custom MATLAB script where ΔF = F-F0 and F0 is the mean fluorescence over 2 s before sucrose presentation. In Fig. 3m, change in dLight3.8 signal in gamma2 and gamma5 mushroom body compartments was computed using ΔF/F = (F − F0)/F0, where F0 is the mean fluorescence over 2 s before the first sucrose presentation. In Fig. 3n, F0 was calculated as the mean fluorescence over 2 s before each sucrose presentation, and the ΔF/F values were averaged across sucrose presentations, sessions and flies. All analysis and plotting were performed using custom Python scripts.

##### Fiber photometry data analysis

Photometry data were processed using custom MATLAB and Python scripts. For single-site and optogenetics recordings, ΔF/F was calculated by regressing the 405 nm isosbestic signal onto the 470 nm dLight signal to generate a fitted baseline, then computing (signal − fitted baseline)/fitted baseline. For event-locked responses, perievent time histograms (PSTHs) were constructed and z-scored using baseline periods. In dual-site benchmarking experiments, sessions with large artifacts were excluded, and bleaching was corrected with a baseline fit (PeakUtils). Signals were averaged across animals and binned by event type (e.g., reward, omission, tone–shock).

For rat recordings, ΔF/F was calculated using linear regression to fit the 430nm isosbestic to the 490nm ligand signal. Traces were z-scored within session. Reward-aligned analyses included baseline normalization across sessions, with linear mixed-effects models used to compare peak amplitudes across reward history conditions (MixedLM, statsmodels). Regressions across trial-back reward history were computed using OLS regression, with Bonferroni correction applied for multiple comparisons at each timepoint.

In the motor timing task, trials were filtered to include only engaged behavior (based on licking patterns). Cue-aligned dLight signals were analyzed, and linear regression was performed to predict post-lick ΔF/F (0.35–0.5 s after first lick) from reward presence and lick timing.

##### Statistical analyses

Statistical analyses were performed using GraphPad Prism, MATLAB, R, or Python. Specific statistical tests (e.g., Student’s t-test, one-way or two-way ANOVA, linear regression, linear mixed-effects models) are described in the text or figure legends. All tests were two-tailed unless otherwise noted. Data are presented as mean ± s.e.m. or mean ± s.d. as indicated. No statistical methods were used to predefine sample size, but sample sizes are similar to those in prior studies. Data distribution was assumed to be normal but not formally tested. No animals or data points were excluded unless otherwise specified. Experimenters were blinded to treatment conditions during analysis when applicable.

## Supplementary Material

Extended Data Figure 1. Sensor Engineering and *in vitro* Characterization

**a,** Simulated structure using Alphafold of dLight 3.6 consisting of DRD1 (dark blue) and cpGFP module (green). Highlighted amino acid residue changes of dLight 3.6 from parent dLight1.3b, including those in the circularly permutated fluorescent protein (cpFP) linker (pink), Beta barrel loops (light blue), and interface between D1DR and cpFP (orange). **b,** dLight construct schematic with flow through of amino acid changes from parent 1.3b to intermediary 3.5 with sequential changes to 3.6 and intermediary 3.7 with final sequential changes to dLight 3.8. **c,** In vitro HEK T-REx 293 stable cell lines basal fluorescence F0 comparison values, n=25. Significance calculated from unpaired t-test for two group comparisons and two-way ANOVA for multiple-group comparisons. ****p<0.0001. **d,** In vitro HEK T-REx 293 stable cell dopamine dose-response curves. Data were fitted with Hill Equation, n=4 for dLight 3.5 and dLight 3.7, n=5 for dLight 1.3b, dLight 3.6, and dLight 3.8. All data shown as mean ΔF/F0± SD. **e,** beta-arrestin recruitment assay demonstrates that the dLight variants do not recruit beta-arrestin. **f,** cAMP analysis in HEK293 cells shows that the dLight variants behave like mock control. **g,** cAMP analysis in U2OS cells shows that the dLight variants behave like moch controls. **h,** Normalized fluorescence intensity scan taken from HEK T-REx 293 stable cell line of dLight3.6 and dLight3.8 under baseline and saturated conditions to determine isosbestic point, dLight 3.6 402nm and dLight 3.8 420nm. Max intensity for dLight 3.6 and dLight 3.8 490nm, n=3. **i,** Normalized emission spectra taken from HEK T-REx 293 stable cell line of dLight3.6 and dLight3.8 under baseline and saturated conditions, n=4. **j,** Table showing amino acid positions and changes from parent sensor dLight 1.3b compared to GRAB ACH3.0, dLight 3.6 and dLight 3.8 within the DRD1, linker, and cpEGFP regions.

Extended Data Figure 2. Pharmacological Screening *in vitro*.

**a,** Representative images of dLight 3.6 and dLight 3.8 in vitro HEK T-REx 293 stable lines. Fluorescence intensity in response to 150nM DA and signal-to-noise ratio of apo and sat state shown. Scale bars = 50 μm. **b,** Schild dose-shift assay for dLight 3.6 and dLight 3.8 at 0nM, 10nM, 100nM, and 1μM concentrations of DRD1 antagonists (SCH-23390 and SKF-83566) followed by dose response of DA addition, n = 6. All data shown as mean ΔF/F0 ± SD. **c,** Representative images of dLight 3.6 and dLight 3.8 in vitro hippocampal neuronal culture. Fluorescence intensity at 150μM NE and signal-to-noise ratio of apo and sat state shown. Scale bars = 50 μm. **d,** in vitro hippocampal neuronal titration basal fluorescence F0 values, n=15. Significance calculated from unpaired t-test for two group comparisons and one-way ANOVA for multiple-group comparisons. ****p<0.0001. Box plot center line shows the median; box limits show upper and lower quartiles. **e,** in vitro hippocampal neuron culture dopamine and norepinephrine dose-response curves. Data were fitted with Hill Equation, n=4 for dopamine dose-response n=3 for norepinephrine dose response. All data shown as mean ΔF/F0 ± SEM. **f,** Dopamine, norepinephrine, and serotonin dose-response curves for dLight 3.6, dLight 3.8, and dLight 1.3b in vitro HEK T-REx 293 stable lines, n=15. All data shown as mean ΔF/F ± SD. **g,** Schild dose-shift assay for dLight 3.6, dLight 3.8, and dLight 1.3b at 0nM, 10nM, 100nM, and 1μM norepinephrine followed by dose response of DA addition, dLight 3.8 n=10, dLight 3.6 and dLight 1.3b n=15. All data shown as mean ΔF/F0 ± SD.

Extended Data Figure 3. *ex vivo* Slice Screening

**a,** Viral constructs made and used in this study were for general (CAG) or Cre-dependent (DIO) dLight neuronal expression. More viruses were designed to express dLight under the Cre-dependent tTA/TRE vectors as a two in one version instead of a split. Traces are from DLS 20 pulses electrical stimulation (3 mice, each virus in one hemisphere) using dLight1 at 3 weeks. **b,** Images are representative of 100 ms frame taken at different time point of a dLight sample expressed on D1-Cells in DLS starting with: a baseline pre-stimulation, the first peak at the onset of stimulation, the last frame just before the end of the 10Hz, 20 pulses stimulation and the recovery 200ms later. The red arrow on the graphs below demonstrates the location of the frame extracted for each example. **c,** (Top) FSCV traces from the DLS of acute slices under control conditions and following a bath application of MPD at 36.5°C ± 0.5°C, mean ± SEM. (Bottom) Traces extracted from imaging in non-injected wild type animals (Left) and D1-Cre injected with AAV1-Syn-dLight1.3b in control followed by the presence of MPD 30μM. **d,** Dynamic range fold increase for dLight3.6 and dLight3.8 after MPD 30μM bath application when normalized to previous version dLight1.3b upon D1-Cre and DAT-Cre sensor expression. Box plot center line shows the median; box limits show upper and lower quartiles. **e,** FSCV traces for DAT-Cre and D1-Cre animals pre and post MPD application. dLight mean ΔF/F0 ± SEM traces at p1P and 20P for samples benchmarked in Figure 1G.

Extended Data Figure 4. Novel Viral Capsids

**a,** Example image averages at baseline and during the peak of a 20 pulses stimulus of dLight3.8 expression in DLS of wild type animals 6 weeks post IV injection using either the PhP.eB or CAP-B10 viral capsid with a CAG promoter. **b,** Corresponding analysis of systemic dLight3.8 delivery analysis traces in DLS (PhP.eB n=12, B10 n=31), PFC (PhP.eB n=3, B10 n=14) and HPC (PhP.eB n=3, B10 n=18). Mean ± SEM for traces in Fig. 2e after 6 weeks comparing the obtained peak amplitude by each using PhP.eB or CAP-B10. **c,** 3D reconstructed whole mouse brain following clearing using IMARIS showing age matched control (no injection) and CAP.B10 dLight 3.8 systemic injection expression pattern. Scale bars 2000um and 3000um respectively. **d,** Representative confocal images of CAP.B10 expression pattern in motor cortex (MC), ventral striatum (VS), and CA1/dentate gyrus (DG) of hippocampus. Scale bars 100um. **e,** Representative lightsheet coronal sections and signal to noise ratio aligned with Allen mouse brain coronal atlas of prefrontal cortex (PFC), dorsolateral striatum (DLS), hippocampus (HPC), and dorsal raphe nucleus (DRN) containing slices.

Extended Data Figure 5. Individual optogenetic stimulations in mouse

**a,** Whole session traces of the mouse optogenetic stimulation experiment at 20Hz stimulation frequency. Red vertical lines denote stimulation times. Green trace shows the dopamine sensor and the blue trace shows the isosbestic control.

Extended Data Figure 6. Dual-fiber photometry in mouse prefrontal cortex and nucleus accumbens during a classical fear conditioning paradigm

**a,** Mean ΔF/F0 ± SEM traces across all animals and trials for the three-day classical fear conditioning paradigm. **b,** Full-width half-maximum comparison of the duration of the shock-response transient shows the signal duration in prefrontal cortex is significantly longer than in nucleus accumbens. Box plot center line shows the median; box limits show upper and lower quartiles. **c,** Single-trial heatmaps of the mean ΔF/F0 demonstrating how the signal changes on a trial-to-trial basis.

Extended Data Figure 7. Mouse *in vivo* mPFC dopamine dynamics during exposure to stimuli with negative, neutral, and positive valence

**a,** (Top) Schematic of dLight expression and fiber placement in the mouse prefrontal cortex. (Bottom Left) Trial average of CS-US evoked and (Bottom Right) CS0 evoked dLight fluorescence during the fear conditioning session. **b,c,** Evolution of **b,** CS evoked and **c,** CS0 evoked average fluorescence across the trials of the fear conditioning session (dLight3.6 n= 4 mice; dLight3.8 n= 4 mice; dLight1.2 n = 3 mice for **b,c**). **d,** Representative heatmaps from a single animal of each variant aligned around tone onset. Though 1.2 variant displayed minimal tone-evoked transients, 3.6 and 3.8 had clear responses observable on a trial-by-trial basis. **e,** Representative heatmaps from a single animal of each variant aligned around the onset of lick bouts for a sucrose solution, demonstrating clear dopamine transients evoked during consumption of an appetite stimulus. **f,** (Left) Group data showing average fluorescence activity over time aligned to tone onset for each of the four variants. (Right) Area under the curve analysis of 5s post-stimulus demonstrating that dLight 3.6 displayed a greater magnitude response to tone compared to the 1.2 and 3.8 variants (one-way ANOVA, F(3, 186) = 15.49, p<0.0001; Tukey’s multiple comparisons test: 3.6 vs 1.2: p<0.0001, 3.6 vs 3.8: p<0.0001, all other pairwise comparisons: p>0.05). **g,** (Left) Group data showing average fluorescence activity over time aligned to lick bout onset for each of the four variants. (Right) Area under the curve analysis of 5s post-stimulus demonstrating that dLight 3.6 displayed a greater magnitude response to sucrose compared to the 1.2 and 3.8 variants (one-way ANOVA, F(3, 281) = 31.467, p<0.0001; Tukey’s multiple comparisons test: 3.6 vs 1.2: p<0.0001, 3.6 vs 3.8: p<0.0001, all other pairwise comparisons: p>0.05). (dLight3.6 n= 5 mice; dLight3.8 n= 4 mice; dLight1.2 n = 5 mice for **f,g**).

Extended Data Figure 8. Dual-fiber photometry in rat prefrontal cortex and dorsomedial striatum during a twoarm bandit task

**a,** Task schematic. **b,** Representative histology showing dLight3.8 sensor expression and fiber track. **c,** Plot of the probability of animals choosing the high reward port relative to the time of the block switch demonstrating that animals adapt their choices to the high reward port shortly after a block switch. **d,** Representative fiber photometry traces in dorsomedial striatum (top) and prefrontal cortex (bottom) during different periods in the task – cues followed by reward collection and cues where no reward is collected. **e,** Trial-averaged traces to the cue onset and reward delivery in dorsomedial striatum (top) and prefrontal cortex (bottom). **f,** Comparison of the response amplitude (top) and full-width half-maximum (bottom) in dorsomedial striatum versus prefrontal cortex to the cue (left) and reward delivery (right). Box plot center line shows the median, and the box limits are the 1st and 3rd quartile. Connected dots show averages for each subject. n = 4 rats.

Extended Data Figure 9. Single-trial analyses during motor timing task

**a,** Schematic for recording NAc DA dynamics with fiber photometry (n=4). **b,c,** Learning curves for all animals with NAc photometry recording across delay training and expert sessions showing that as the delay period increases, animals gradually learn to lick later. Delay duration (b) and mean time to first lick (c) across sessions is shown. Thin lines, individual animals, thick lines, mean across animals. **d,** Average NAc DA response aligned to cue onset in an example animal during learning (left) and expert sessions (right). Colors indicate normalized session numbers. Post-cue NAc DA response decreases during delay training and increases again in expert sessions. Quantification in Fig 6f. **e,** Mean post-cue NAc DA response per session. Thin lines, individual animals, thick lines average across animals. Cue response shows a U-shaped curve, decreasing during delay training and increasing again in expert mice. **f,** Average post-cue NAc DA response in sessions grouped by early delay training (delay duration 0.1~0.7s), late delay training (delay duration 0.7~1.3 s), and expert (1.5 s fixed delay). **g,** Least squares regression slope of single-trial post-cue NAc DA response on lick time per session. Thin lines, individual animals, thick lines average across animals. **h,** Regression slope in sessions grouped as in (f). Cue response becomes less predictive of upcoming lick timing as animals learn to lick later. **i,** Average NAc DA response aligned to lick in an example animal in rewarded (left) and unrewarded trials (right). Colors indicate normalized session numbers. **j,** Mean post-lick NAc DA response in rewarded (blue) and unrewarded (black) trials per session. Thin lines, individual animals, thick lines average across animals. **k,** Average post-lick response in rewarded trials during delay training and in expert sessions. Post-lick NAc DA response increases in rewarded trials during both delay training and experts, consistent with positive reward prediction error. **l,** Average post-lick response in unrewarded trials during delay training and in expert sessions. Post-lick NAc DA response decreases in unrewarded trials, and the decrease is higher later in training, suggesting that the negative reward prediction error emerges later in learning. **m,** Schematic for recording DLS DA dynamics with fiber photometry (n=4). **n,o,** Learning curves for all animals with DLS photometry recording across delay training and expert sessions showing that as the delay period increases, animals gradually learn to lick later. Delay duration (n) and mean time to first lick (o) across sessions is shown. Thin lines, individual animals, thick lines, mean across animals. **p,** Average DLS DA response aligned to cue onset in an example animal during learning (left) and expert sessions (right). Colors indicate normalized session numbers. Post-cue DLS DA response decreases during early delay training and does not increase in expert sessions, unlike the in NAc (i). **q,** Mean post-cue DLS DA response per session. Thin lines, individual animals, thick lines average across animals. **r,** Average DLS cue response in sessions grouped by early delay training (delay duration 0.1~0.7s), late delay training (delay duration 0.7~1.3 s), and expert (1.5 s fixed delay). **s,** Least squares regression slope of single-trial post-cue DLS DA response on lick time per session. Thin lines, individual animals, thick lines average across animals. **t,** Regression slope in sessions grouped as in R. DLS cue response is weakly predictive of upcoming lick time only during learning but not in expert sessions. **u,** Average DLS DA response aligned to lick in an example animal in rewarded (left) and unrewarded trials (right). Colors indicate normalized session numbers. **v,** Mean post-lick DLS DA response in rewarded (blue) and unrewarded (black) trials per session. Thin lines, individual animals, thick lines average across animals. **w,** Average DLS post-lick response in rewarded trials during delay training and in expert sessions. **x,** Average DLS post-lick response in unrewarded trials during delay training and in expert sessions. Post-lick DLS DA response increases in rewarded trials during both delay training and experts, consistent with positive reward prediction error. However, post-lick response decreases in unrewarded trials more early in training, in contrast to changes in the NAc with training. All box plots show center line as the median; box limits show upper and lower quartiles.

Extended Data Figure 10. FLIM and FLIPR

**a,** Mice were injected with AAV9-CAG-dLight3.8 at the tail of the striatum. Representative intensity (left) and lifetime (right) heatmaps in a given field of view. Intensity image is at 1x zoom, while lifetime images are all collected at 10x zoom. Lifetime images show comparison of per pixel average fluorescence lifetime with a wash on of D1 antagonist (SCH23390, 10 uM) or D1 agonist (SKF81297, 1 uM). **b,** Comparison of average fluorescence lifetime of per field of view with a wash on of D1 antagonist or D1 agonist. Unpaired, two-sample, two-way t-test; p = 1.56 × 10^−15. **c,** Mice were injected with AAV9-CAG-dLight3.8 and an optical fiber implant was targeted to the Nucleus accumbens core. After recovery, mice received chocolate pellets in a neutral box. Simultaneously, fluorescence intensity and fluorescence lifetime were measured using FLIPR at 10khz and consumption behavior was video recorded. **d,** Delta fluorescence intensity (left top) and fluorescence lifetime (left bottom) was recorded in response to food pellet consumption for separate hemispheres (average trace per hemisphere, n = 6 hemispheres). Average delta fluorescence intensity (right top) and fluorescence lifetime (right bottom) changes in response to food pellet consumption across hemispheres (n=6).

Supplementary Figure 1. Sequence Alignment

Supplementary Figure 2. Histological Verification for Dual-Fiber Photometry Recordings in NAc and PFC

Supplementary Table 1. Key Reagent and Resources Table

Supplementary Table 2. Properties in hippocampal neuronal cultures

Supplementary Table 3. Effective Dopamine Response Under Antagonist

Supplementary Table 4. Sensor properties in HEK T-Rex cells

Supplementary Table 5. Effective Dopamine Response Under Norepinephrine

Supplementary Table 6. dLight sensor properties when expressed in various ways and locations

Supplementary Files

This is a list of supplementary les associated with this preprint. Click to download.

- SupplementaryFigures.pdf
- SupplementaryTable1.xlsx
- SupplementaryTable2.xlsx
- SupplementaryTable3.xlsx
- SupplementaryTable4.xlsx
- SupplementaryTable5.xlsx
- SupplementaryTable6.xlsx
- ExtendedDataFigure1final.pdf
- ExtendedDataFigure2final.pdf
- ExtendedDataFigure3final.pdf
- ExtendedDataFigure4final.pdf
- ExtendedDataFigure5final.pdf
- ExtendedDataFigure6final.pdf
- ExtendedDataFigure7final.pdf
- ExtendedDataFigure8final.pdf
- ExtendedDataFigure9final.pdf
- ExtendedDataFigure10final.pdf

## Data Availability

The following public dataset was used to support this study: Allen Brain Atlas ISH data (https://mouse.brain-map.org/). All source data present in this manuscript are available from https://github.com/tianlab-mpfi/dLight3_Dopamine_Sensors/tree/main/Source%20Data.

## RESOURCE AVAILABILITY

### Materials Availability & Correspondence

The following plasmids and viral constructs have been deposited in Addgene and UNC neurotools: CAG-dLight3.6, CAG-dLight3.8, CAG-DIO-dLight3.6, CAG-DIO-dLight3.8, hSyn-tTA-TRE-dLight3.6, hSyn-tTA-TRE-dLight3.8, hSyn-tTA-TRE-DIO-dLight3.6, and hSyn-tTA-TRE-DIO-dLight3.8.

The Drosophila 20xUAS-dlight3.8 lines are available on request from the Bidaye lab and will be soon available through Bloomington Drosophila Stock Center.

Lead contact: lin.tian@mpfi.org

## CODE AVAILABILITY

All custom MATLAB/Python codes are available from https://github.com/tianlab-mpfi/dLight3_Dopamine_Sensors/tree/main/Analysis%20Code.
